# A nine-gene diagnostic model for IgA nephropathy based on multi-cohort machine learning: integrating gene expression and immunohistochemical validation

**DOI:** 10.1080/0886022X.2026.2637355

**Published:** 2026-03-09

**Authors:** Yating Ge, Xiao Jiang, Jinlian Shu, Xueqi Liu, Yonggui Wu

**Affiliations:** a Department of Nephrology, The Second People’s Hospital of Hefei, Hefei Hospital Affiliated to Anhui Medical University, Hefei, Anhui, China; b The Department of Nephrology, The First Affiliated Hospital of Anhui Medical University, Hefei, Anhui, PR China; c Center for Scientific Research of Anhui Medical University, Hefei, Anhui, China

**Keywords:** IgA nephropathy, machine learning, gene expression profiling, biomarkers, precision medicine, immunohistochemistry

## Abstract

IgA nephropathy (IgAN) is the most common primary glomerulonephritis, requiring improved diagnostic tools. We analyzed three cohorts (GSE37460, GSE93798, and GSE115857 are internal validation cohorts) using gene set enrichment analysis on 7751 pathways. A machine learning model was developed and externally validated in multi-cohort gene expression data (external validation cohorts are GSE99339, GSE116626, and GSE104948). Additionally, immunohistochemistry was performed to validate the expression of key biomarkers and the presence of functionally active immune cells. We developed and validated a multi-cohort machine learning diagnostic model. The selected two-step glmBoost + Enet [alpha = 0.4] model achieved high concordance in GSE37460 (κ = 0.704, *p* < 0.001), GSE93798 (κ = 0.486, *p* < 0.001), and GSE115857 (κ = 1.000, *p* < 0.001). Applying the same model to the external validation cohorts demonstrated strong diagnostic accuracy with AUCs of 0.938 (GSE99339), 0.871 (GSE116626), and 0.926 (GSE104948); the corresponding Kappa statistics were κ = 0.699 (*p* < 0.001), 0.443 (*p* = 0.018), and 0.615 (*p* = 0.023). Nine genes were identified as significant for the diagnosis of IgAN, and *HLA-DRA* and *VASH1* emerged as robust biomarkers across the cohorts (all *p* < 0.05). Additionally, immunohistochemistry validation demonstrated a marked increase in HLA-DRA and VASH1 expression in IgAN patients. Immunofluorescence staining indicated a greater presence of CD4 + HLA-DRA+ functionally active/activated CD4+ T cells in IgAN tissues than in controls. This study delivers a reproducible 9-gene machine-learning classifier for precise IgA nephropathy diagnosis and highlights HLA-DRA and VASH1 as promising biomarkers.

## Graphical Abstract

**Figure:**
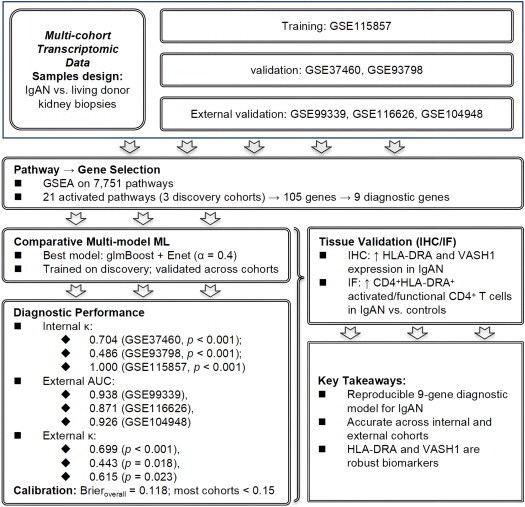

IgA nephropathy (IgAN) is a kidney disease caused by immune deposits that damage the kidney filters (glomeruli). It is one of the most common causes of kidney failure worldwide, but diagnosis still requires a kidney biopsy. In this study, we used advanced computer models (machine learning) to find gene patterns that can help identify IgAN from kidney tissue samples. We analyzed data from six international research databases and tested over 80 model combinations. From this, we discovered a set of nine genes that accurately distinguished IgAN from other kidney diseases. The results were confirmed using independent patient groups and laboratory staining of kidney tissue. This research may help create noninvasive genetic or blood tests in the future, reducing the need for biopsy and improving early diagnosis for people with suspected IgA nephropathy. The graphical abstract for this study has been provided as Supplementary Figure 1.

## 1. Introduction

Immunoglobulin A nephropathy (IgAN) is a clinical syndrome characterized by pathological immune features in which IgA is deposited predominantly in the mesangial area of the renal glomerulus. It is the most common chronic glomerular disease worldwide, with a global incidence rate exceeding 2.5 per 100,000 people [1,2]. This disease is more common in older children and young adults and is often preceded by symptoms such as upper respiratory tract infections. The clinical manifestations of IgAN vary, ranging from mild urinary abnormalities to rapidly progressive glomerulonephritis [3,4]. Approximately 30% of patients develop end-stage renal disease, necessitating renal replacement therapy after approximately 20 years [5].

The exact mechanism of IgAN remains unclear, and a definitive diagnosis requires a kidney biopsy. It is generally believed that IgAN is caused by immune-mediated damage. The prevailing multiple-hit theory suggests that excessively produced aberrantly glycosylated IgA1 (Gd-IgA1) acts as an antigen, eliciting the production of specific autoantibodies. Subsequently, circulating immune complexes are formed and eventually deposit in the mesangium of the renal glomeruli, leading to chronic inflammation and kidney damage [6]. Despite progress in elucidating the pathophysiology of IgAN, there is still an absence of targeted therapeutic options. The current management of IgAN involves broad-based strategies common to all chronic glomerular diseases: attenuation of proteinuria, administration of renin-angiotensin system blockers, and meticulous blood pressure regulation. In patients who exhibit persistent proteinuria or deteriorating renal function despite conservative measures, a body of clinical research advocates the administration of corticosteroids to ameliorate proteinuria in IgAN patients. However, recent studies have cast doubt on the efficacy of this approach for the majority of patients, highlighting the significant risks of severe adverse effects, particularly infections [7,8]. Hence, it is imperative to identify biomarkers that can aid in the diagnosis of IgAN, facilitate the discovery of innovative therapeutic interventions, and assist in the assessment of patient prognosis.

In recent years, gene expression profiling technologies have been widely employed to identify new biomarkers and potential molecular mechanisms underlying various diseases. For instance, Berthier et al. [9] conducted gene expression profiling analysis on renal glomerular tissues from 27 IgAN patients, while Liu et al. [10] performed gene expression profiling analysis on renal glomerular tissues from 20 patients. In the current investigation, we included several cohorts consisting of patients with IgAN and healthy controls. Our initial step involved the identification of activated signaling pathways within the IgAN cohorts. Subsequent to this pathway identification, we aimed to pinpoint key genes within these pathways. Using machine learning techniques, we constructed a diagnostic model for IgAN anchored by these key genes. This integrative approach not only enhances our understanding of the disease mechanism but also aids in the discovery of reliable biomarkers for its diagnosis. Additionally, by incorporating data relevant to glomerular function, we sought to elucidate the characteristics of immune cell infiltration, thereby paving the way for potential therapeutic strategies for IgAN. This study exemplifies how artificial intelligence, particularly machine learning, can transform nephrology by improving noninvasive diagnosis of IgA nephropathy.

## 2. Materials and methods

### 2.1. Basic information of the enrolled cohorts

We downloaded all the samples from the GEO database, which included gene expression profiles and clinical information. The total number of individuals across all cohorts was 207 IgAN patients and 56 living donors (LDs). All the IgAN samples were validated by a pathologist as described in the literature.

GSE115857 served as the training cohort, while GSE37460 and GSE93798 were utilized for the validation of the diagnostic model. The GSE104948, GSE116626, and GSE99339 datasets served as the external validation cohorts. Table 1 lists the numbers of IgAN patients and LDs in each cohort: GSE104948 included 27 IgAN patients and 3 LDs, GSE115857 included 55 IgAN patients and 7 LDs, GSE116626 included 52 IgAN patients and 7 LDs, GSE37460 included 27 IgAN patients and 9 LDs, GSE93798 included 20 IgAN patients and 22 LDs, and GSE99339 included 26 IgAN patients and 8 LDs.

**Table 1.** Basic information of the enrolled cohorts.

	IgAN	LD	Overall	Source
	(*N* = 207)	(*N* = 56)	(*N* = 263)	
GSE104948	27	3	30	https://www.ncbi.nlm.nih.gov/geo/query/acc.cgi?acc=GSE104948
GSE115857	55	7	62	https://www.ncbi.nlm.nih.gov/geo/query/acc.cgi?acc=GSE115857
GSE116626	52	7	59	https://www.ncbi.nlm.nih.gov/geo/query/acc.cgi?acc=GSE116626
GSE37460	27	9	36	https://www.ncbi.nlm.nih.gov/geo/query/acc.cgi?acc=GSE37460
GSE93798	20	22	42	https://www.ncbi.nlm.nih.gov/geo/query/acc.cgi?acc=GSE93798
GSE99339	26	8	34	https://www.ncbi.nlm.nih.gov/geo/query/acc.cgi?acc=GSE99339

### 2.2. Dismissal of batch effects

Batch effects are the nonbiological differences between two or more datasets. To eliminate the bias caused by batch effects in this study and make the transcription profiles in the three GEO cohorts more similar, the ComBat algorithms in the “sva” package were used to remove the batch effects between these three GEO-sourced cohorts. Normalization, feature selection, and batch correction were performed within each training fold prior to validation/testing to avoid information leakage.

### 2.3. Calculation of the scores of signaling pathways

Gene set enrichment analysis (GSEA) is a computational method that determines whether a set of genes shows statistically significant differences between two groups. We employed GSEA to first compare the diverse activated signaling pathways between IgAN patients and LDs. The background file of molecular signature gene sets was downloaded from MSigDB, C5: Biological Process, with a total of 7,751 gene sets[11,12].

### 2.4. Predictive model generated from machine learning-based integrative approaches

To establish a comparative multi-model framework characterized by high precision and robust performance in differentiating IgAN patients from healthy individuals, we synthesized the capabilities of nine machine learning algorithms and eighty of the 101 algorithmic combinations produced valid results suitable for comparative evaluation. The ensemble of algorithms comprised elastic net (Enet), lasso, ridge, stepwise generalized linear models (both forward and backward selection), gradient boosting machines (glmBoost), generalized boosted regression modeling (GBM), linear discriminant analysis (LDA), and naive Bayes. The protocol for signature generation was as follows: (a) the most significantly activated biological pathways in IgAN patients across three GEO datasets, namely, GSE115857, GSE37460, and GSE93798, were identified; (b) the selected genes from the most activated pathways were subjected to 80 algorithmic combinations; (c) each model was trained on the GSE115857 dataset and validated on the GSE37460 and GSE93798 cohorts, followed by external testing on the GSE104948, GSE116626, and GSE99339 datasets, which were not utilized during the pathway filtration process; and (d) for every individual model, the area under the receiver operating characteristic curve (AUC) was computed across all the cohorts involved in the study.

For model optimization, hyperparameters for each algorithm (e.g. α and λ for Enet/Lasso/Ridge, number of boosting iterations and learning rate for GBM, and shrinkage rate for glmBoost) were tuned *via* 10-fold cross-validation within the training cohort using grid search. Model stability was assessed by averaging performance over 100 random resampling iterations to minimize overfitting. To avoid data leakage, all preprocessing steps (normalization, scaling, and feature selection) were performed independently within each training fold before model fitting. Validation and external datasets were held out until the final evaluation stage and were not used in any way for model construction or parameter tuning.

### 2.5. Infiltration of immune cells

To gauge the unique immune infiltration within a population, we employed single-sample gene set enrichment analysis (ssGSEA), which ascertains an enrichment score reflecting the extent of absolute enrichment of an immune cell-associated gene set within each individual sample of the dataset in question. Normalized enrichment scores were computed for each immunological category [13]. The ssGSEA analyses were conducted utilizing the GSVA package in R.

### 2.6. Patient enrollment and tissue collection

IgAN patients and controls were enrolled from the nephrology department at our institution. The inclusion criteria for IgAN patients were biopsy-proven IgAN with available clinical data, while controls were selected from individuals who underwent nephrectomy for reasons other than glomerular disease, ensuring no history of renal pathology.

### 2.7. Immunohistochemistry and immunofluorescence staining

IgAN patients and controls were selected for the validation of critical genes or immunocytes (Table 2). For immunohistochemistry (IHC), paraffin-embedded sections were deparaffinized in xylene and rehydrated through graded ethanol solutions. Antigen retrieval was performed using a microwave in either EDTA buffer (pH 9.0) or citrate buffer (pH 6.0). Endogenous peroxidase activity was blocked with 3% hydrogen peroxide. The sections were incubated overnight at 4 °C with primary antibodies against VASH1 (Zenbio, 1:400) and HLA-DRA (Abways, 1:400). After washing, the sections were incubated with an HRP-conjugated secondary antibody (Shanghai Huilan Biotechnology) for 2 h and developed with DAB substrate to visualize positive signals as brown staining. Counterstaining was performed with Harris hematoxylin, followed by dehydration, clearing in xylene, and mounting with neutral resin.

**Table 2.** Clinical features of selected IgAN patients and controls.

Variable	Overall (*N* = 40)^1^	Control (*N* = 8)^1^	IgAN (*N* = 32)^1^	*p*-value^2^
Age, years	39.0 (29.8–45.3)	44.0 (40.0–46.5)	36.0 (28.8–45.3)	0.160
Body Mass Index (BMI), kg/m²	23.6 (21.8–26.5)	23.8 (21.9–28.0)	23.6 (21.8–25.6)	0.467
Systolic Blood Pressure (SBP), mmHg	119.0 (110.0–125.0)	122.5 (118.8 –129.3)	116.0 (109.75–125.0)	0.136
Diastolic Blood Pressure (DBP), mmHg	76.5 (69.5–81.0)	80.5 (72.0–86.0)	76.0 (67.75–81.0)	0.416
Albumin (ALB), g/L	41.7 (38.42– 45.3)	40.85 (38.1– 42.1)	42.5 (38.4– 45.7)	0.499
Blood Urea Nitrogen (BUN), mmol/L	5.05 (4.48–6.10)	5.76 (5.06–5.92)	4.945 (4.42–6.18)	0.370
Serum Creatinine (Scr), µmol/L	79.8 (65.0–99.5)	83.5 (62.75– 110.5)	77.3 (65.9–94.3)	0.778
Estimated Glomerular Filtration Rate (eGFR), mL/min/1.73 m²	103.5 (73.75–115.0)	85.0 (57.0–106.0)	105.0 (80.758–116.0)	0.166
Uric Acid (UA), µmol/L	347.8 (283.2–400.2)	294.0 (241.5– 426.7)	364.0 (292.8–426.5)	0.043
Total Cholesterol (TC), mmol/L	4.07 (3.32–4.75)	4.115 (3.77– 4.45)	4.05 (3.29–4.76)	0.881
Triglycerides (TG), mmol/L	1.60 (1.09–2.08)	1.21 (1.09–1.94)	1.64 (1.08–2.09)	0.774
Low-Density Lipoprotein (LDL), mmol/L	2.58 (2.07–2.87)	2.47 (2.35–2.89)	2.61 (1.99–2.87)	> 0.999
24-Hour Urine Total Protein (24h UTP), g/24h	0.58 (0.29–0.95)	/	0.58 (0.29–0.95)	/
Urine Transferrin (Urine TRF), mg/24h	23.65 (10.65–53.81)	/	23.65 (10.65–53.81)	/
Urine Beta-2 Microglobulin (Urine β2MG), mg/L	0.165 (0.09–0.358)	/	0.165 (0.09–0.358)	/
Urine Alpha-1 Microglobulin (Urine α1MG), mg/L	9.935 (5.158–13.15)	/	9.935 (5.158–13.15)	/
Urine Albumin to Creatinine Ratio (Urine A/C), mg/g	345.95 (169.98–739.33)	/	345.96 (169.98–739.33)	/
Sex, n (%)				0.690
Female	16 (40)	4 (50)	12 (38)	
Male	24 (60)	4 (50)	20 (63)	

^1^ Median (IQR) or frequency (%).

^2^ Wilcoxon rank sum test; Wilcoxon rank sum exact test; Fisher’s exact test.

For immunofluorescence(IF), paraffin-embedded sections were similarly deparaffinized and rehydrated, followed by antigen retrieval in EDTA buffer (pH 9.0) or citrate buffer (pH 6.0). Endogenous peroxidase activity was blocked with 3% hydrogen peroxide for 25 min. The sections were blocked with 3% BSA for 10 min and then incubated overnight at 4 °C with primary antibodies against CD4 (ZSGB-BIO, 1:200) and HLA-DRA (Abways, 1:200). After washing in PBS, the sections were incubated for 1 h with species-specific secondary antibodies. For CD4 and HLA-DRA dual staining, primary antibodies were applied simultaneously, followed by appropriate fluorescent secondary antibodies. Fluorescent signals were developed, and the sections were counterstained with DAPI for 10 min. Sections were mounted with anti-fade medium and examined using a Nikon inverted fluorescence microscope.

### 2.8. Reporting guidelines and reproducibility

To enhance transparency and reproducibility, Supplementary Figure 2 (workflow diagram) summarizes the end-to-end pipeline, including data sources, preprocessing, feature selection, model development and validation, and IHC/IF experimental validation leading to the final outputs.

This study adheres to established reporting standards for diagnostic model development, AI/ML research, and biomarker validation. We followed TRIPOD for transparent reporting of prediction model development and validation [14] (Supplementary Table 1); MINSEQE for ensuring the reproducibility of gene-expression profiling (including platforms/annotations, preprocessing, batch correction, and code/environment disclosure) [15] (Supplementary Table 2); and STROBE for observational reporting elements [16] (Supplementary Table 3). The completed checklists are provided as Supplementary Materials.

### 2.9. Statistics

All the statistical analyses were performed with R software (version 4.2.2). Fisher’s exact test was employed for the analysis of categorical data, while Pearson’s correlation coefficient was utilized for the examination of continuous variables. The identified pathways were visualized in a heatmap generated by the “pheatmap” package in R. The kappa statistic served as a quantitative measure of the concordance between the predicted and actual subtypes. For statistical comparisons involving more than two groups, the Kruskal–Wallis test was applied, whereas the Wilcoxon rank-sum test was used for comparisons between two groups [17]. For two-group comparisons, we used parametric t-tests when normality was satisfied; otherwise, we used the Mann-Whitney U test. A two-tailed *p* value of less than 0.05 was considered to indicate statistical significance.

## 3. Results

### 3.1. Normalization and comparative analysis of signaling pathway activation in IgAN across multiple cohorts

**Figure 1.**
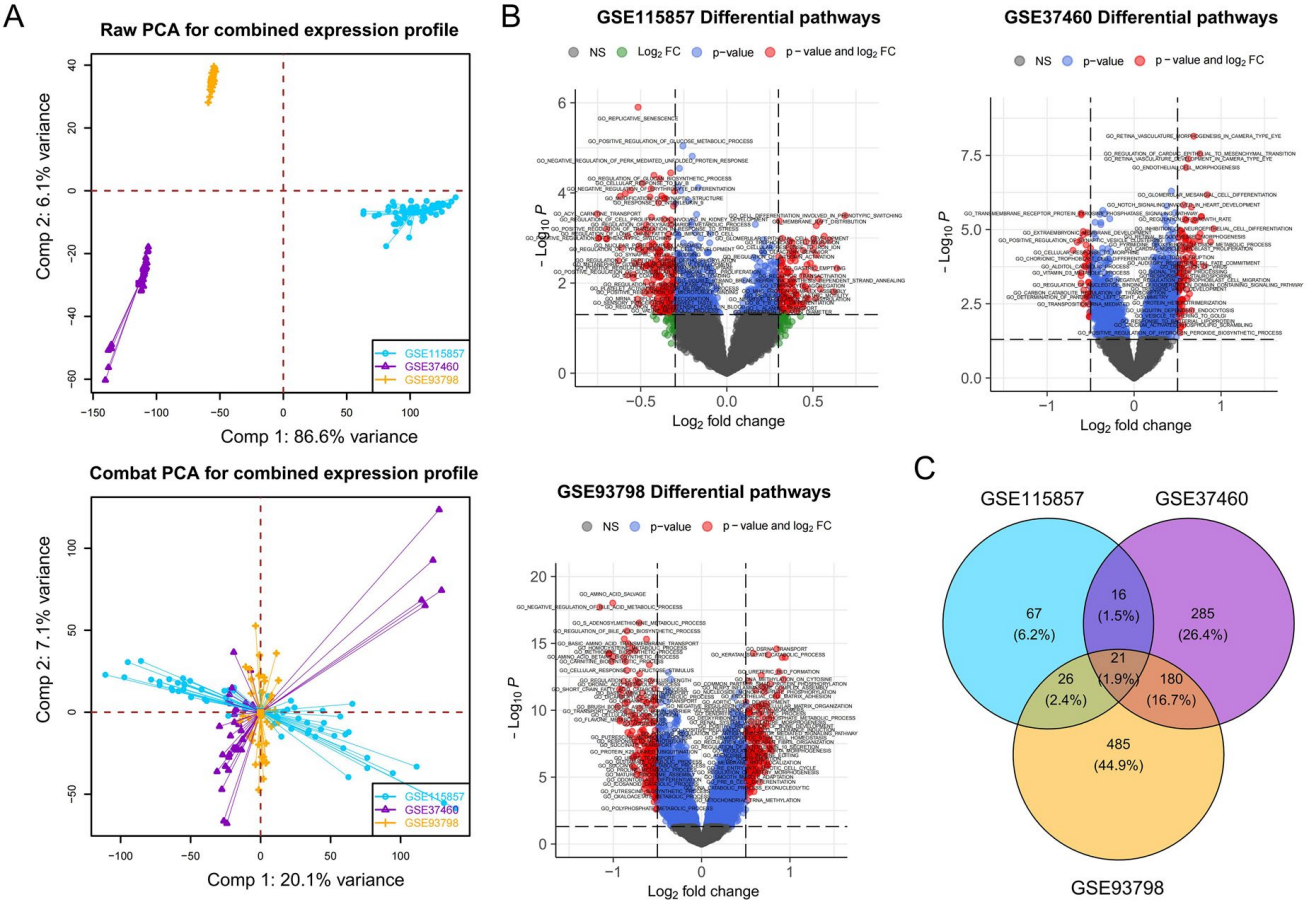
Batch normalization and gene expression pathway analysis in IgA nephropathy (IgAN) patients versus healthy donor controls across GEO cohorts (GSE115857, GSE37460, GSE93798). (A) PCA plots showing variation in gene expression before (upper plot) and after (lower plot) batch normalization across the GSE115857, GSE37460, and GSE93798 cohorts. (B) Volcano plots for each cohort displaying differentially activated pathways in IgAN patients. (C) Venn diagram representing common significantly activated pathways in IgAN patients among the cohorts, with 21 pathways consistently altered.

**Figure 2.**
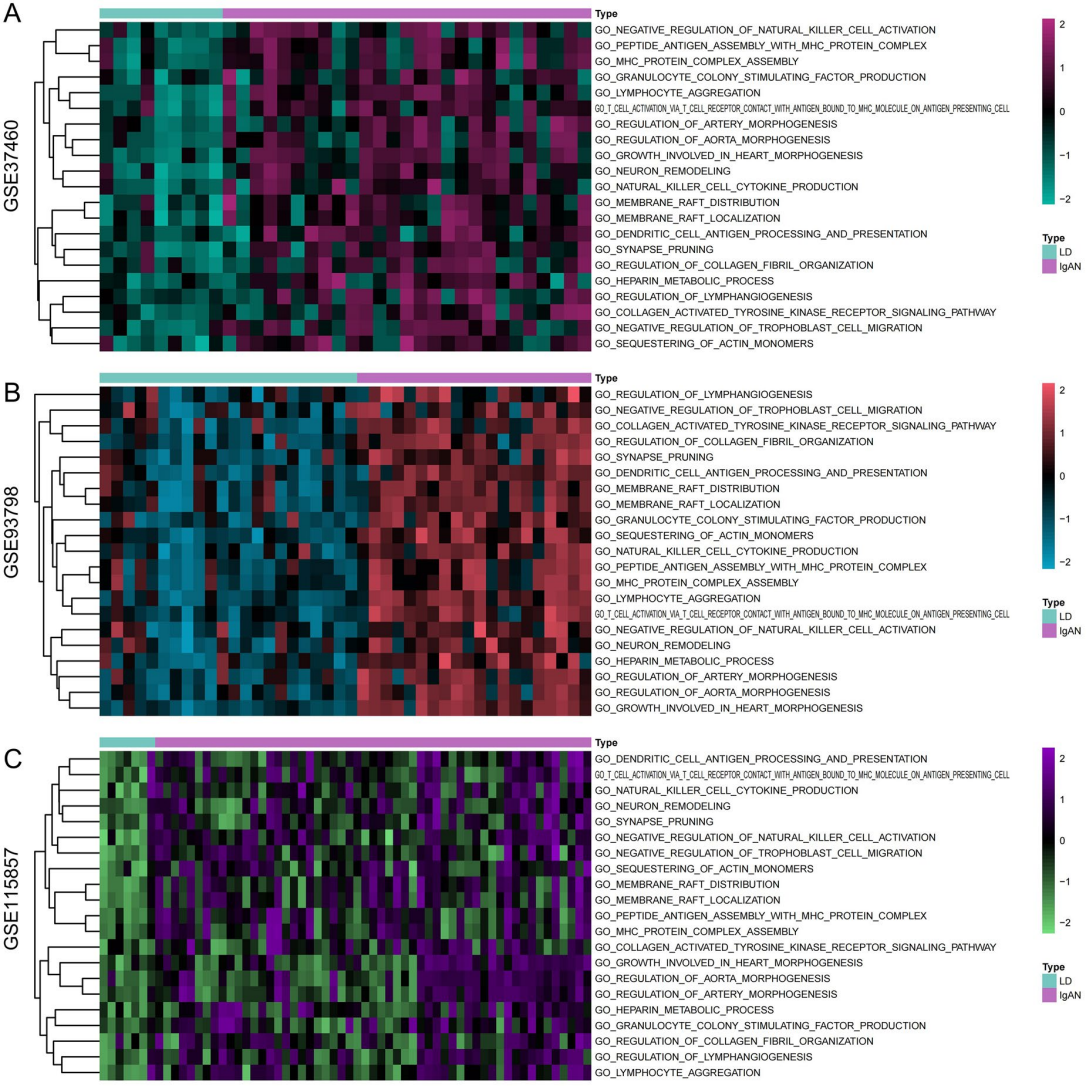
Heatmap representation of the 21 distinct signaling pathways significantly activated in IgAN patients. (A) GSE37460 cohort, (B) GSE93798 cohort, and (C) GSE115857 cohort. Each row represents a signaling pathway, and each column represents a sample within the cohort.

We initially included three internal validation cohorts: GSE115857, GSE37460, and GSE93798. Because the cohorts originated from different datasets, we performed batch normalization procedures to eliminate batch effects caused by sequencing operations (Figure 1A). Subsequently, we utilized GSEA (gene set enrichment analysis) to assess the activation levels of different cellular signaling pathways in all included samples. Within each cohort, we compared the signaling pathways with differential activation between IgAN patients and living donors, and signaling pathways exhibiting a log2-fold change greater than 0.3 and a *p* value less than 0.05 were considered to be significantly different. In the GSE115857 cohort, we observed that 130 signaling pathways were significantly activated in IgAN patients; in the GSE37460 cohort, there were 502 significantly activated signaling pathways; and in the GSE93798 cohort, there were 712 activated pathways (Figure 1B). Ultimately, we identified a total of 21 signaling pathways that were significantly activated in IgAN patients based on the consistent results from the three cohorts (Figure 1C). The curation of 21 distinct signaling pathways predominantly involves mechanisms integral to immune modulation, cellular architecture and signaling, developmental biology, metabolic processing, neurodevelopment, and hematopoiesis (Figure 2, Supplementary Table 4).

### 3.2. An IgAN diagnostic model was developed by machine learning approaches

**Figure 3.**
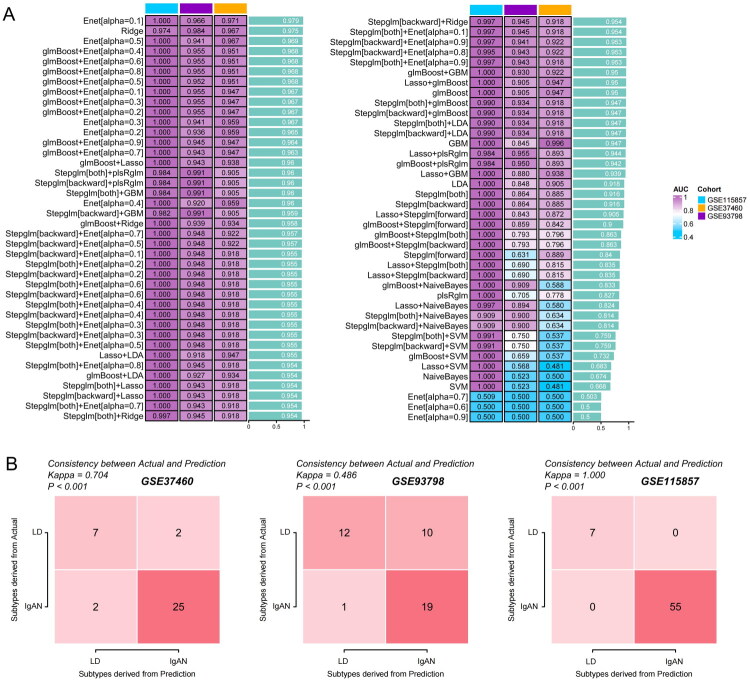
Construction of a machine learning diagnostic model for IgA nephropathy (IgAN) using glmBoost + elastic net al.gorithms and gene expression biomarkers. (A) Heatmap summarizing the AUC values for 80 machine learning model combinations in the three cohorts. (B) Kappa statistic matrices for the glmBoost + Enet[alpha = 0.4] model displaying diagnostic concordance with the actual values for GSE115857, GSE37460, and GSE93798.

The 21 selected signaling pathways comprising 105 unique genes were used as input data. As delineated in the methodology, the construction of the diagnostic model involved 101 different algorithms. Of these, the results of 80 combinational methods were successfully produced, with the remainder potentially unsuitable for the present study due to the limited quantity of input data. The AUC values for each method, as depicted in Figure 3A, indicate that the Enet [alpha = 0.1], Ridge, and Enet [alpha = 0.5] models achieved average AUC values of 0.979, 0.975, and 0.969, respectively. However, an inspection of the genes incorporated within the models revealed that they require 85, 105, and 22 genes, respectively (Supplementary Table 5), which presents a considerable challenge for clinical application. Consequently, we opted for a two-step model, glmBoost + Enet [alpha = 0.4], which not only demonstrated a high AUC value of 0.968 but also necessitated only 9 input genes, including “*CD160*,” “*CX3CR1*,” “*EPHA4*,” “*THBS1*,” “*HLA-DRA*,” “*FARP2*,” “*VASH1*,” “*TMSB4Y*,” and “*RHBDD3*” (Supplementary Table 6). Additionally, the predictive value of this model was corroborated through kappa analysis, which showed significant concordance in GSE37460 (κ = 0.704, *p* < 0.001), GSE93798 (κ = 0.486, *p* < 0.001), and GSE115857 (κ = 1.000, *p* < 0.001), as shown in Figure 3B.

### 3.3. Confirming the diagnostic performance in independent cohorts

**Figure 4.**
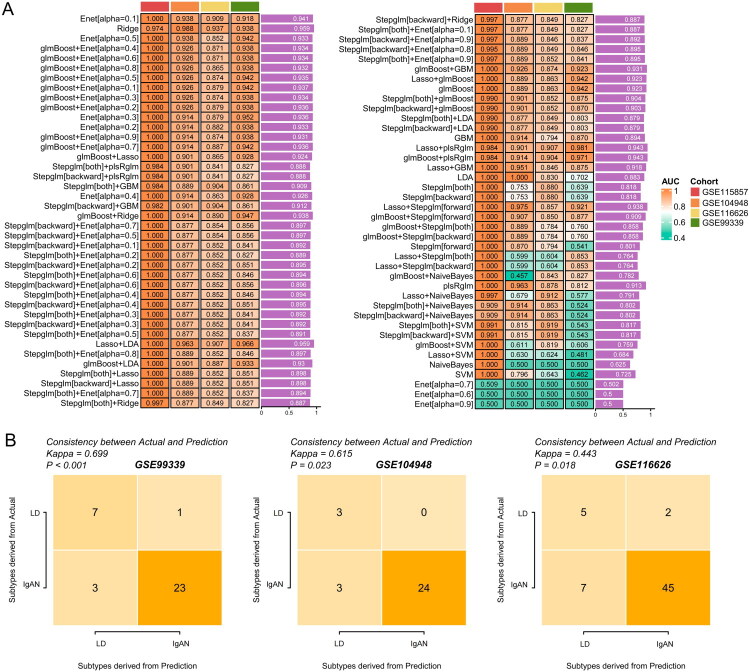
Validation of the IgAN diagnostic model in independent cohorts. (A) Heatmap indicating the AUC values of 80 predictive models in the GSE99339, GSE116626, and GSE104948 cohorts. (B) Kappa statistics for the glmBoost + Enet [alpha = 0.4] model demonstrating high concordance across the independent cohorts.

To ascertain the efficacy of the diagnostic model we developed, we harvested samples from three independent external validation cohorts: GSE99339, GSE116626, and GSE104948. Employing the previously established methodology, we conducted predictive analyses using 80 models across these separate cohorts. Notably, the glmBoost + Enet model, with an alpha set at 0.4, demonstrated exceptional diagnostic precision, as depicted in Figure 4A, yielding the following outcomes: GSE99339 (AUC = 0.938, κ = 0.699, *p* < 0.001), GSE116626 (AUC = 0.871, κ = 0.443, *p* = 0.018), and GSE104948 (AUC = 0.926, κ = 0.615, *p* = 0.023, Figure 4B). These findings suggest that the model’s predictive validity is remarkably robust and holds promise for effective clinical application.

### 3.4. Expression of 9 characteristic genes in IgAN

**Figure 5.**
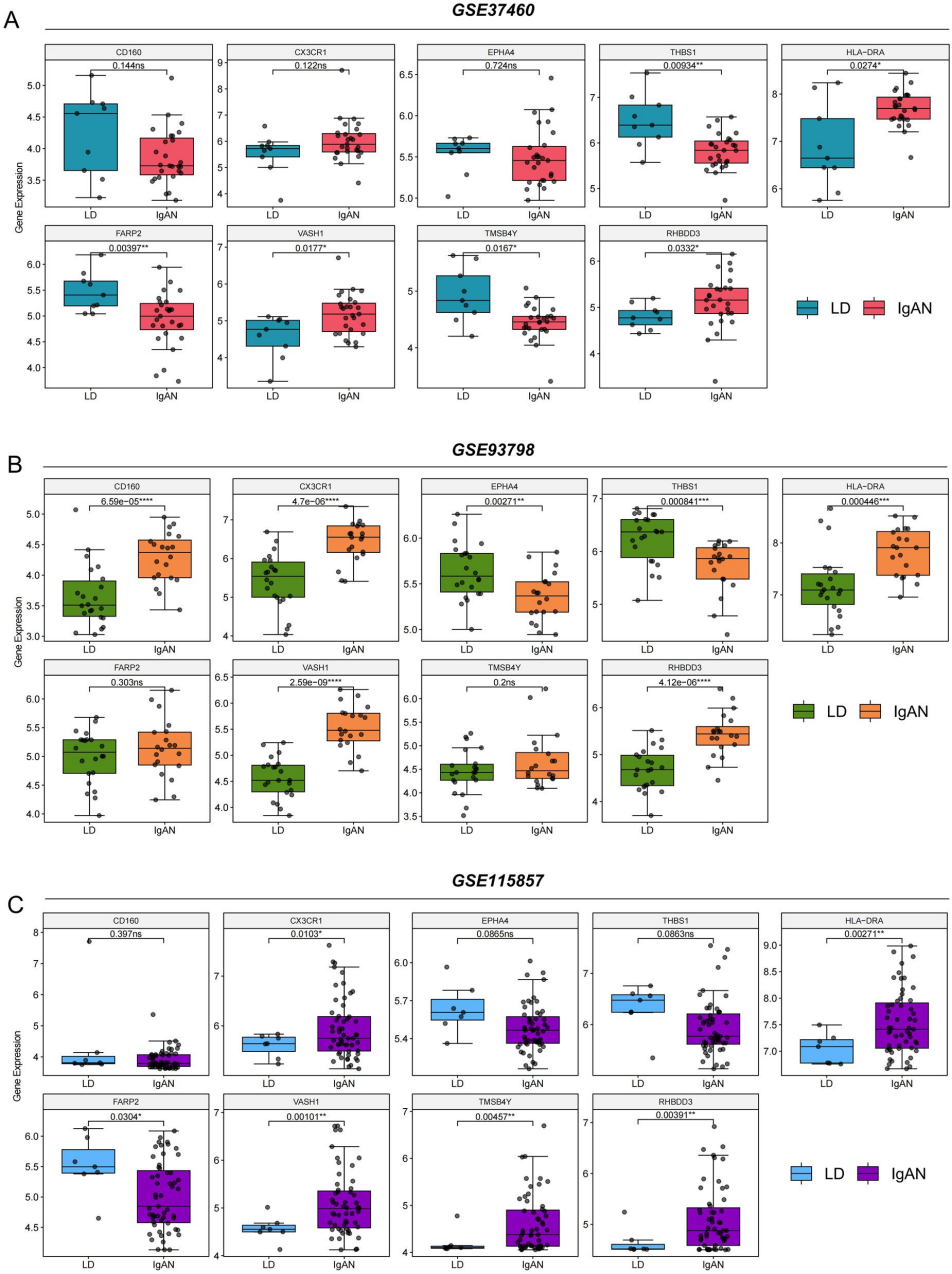
Expression of diagnostic biomarkers (HLA-DRA, VASH1, RHBDD3, etc.) identified by machine learning in IgA nephropathy (IgAN) compared to living donor controls. Box plots illustrating the differential expression of *CD160*, *CX3CR1*, *EPHA4*, *THBS1*, *HLA-DRA*, *FARP2*, *VASH1*, *TMSB4Y*, and *RHBDD3* between IgAN patients and living donors across three cohorts: (A) GSE37460, (B) GSE93798, and (C) GSE115857.

The glmBoost + Enet [alpha = 0.4] model incorporated 9 genes into the diagnostic model for IgAN. To analyze the expression patterns of these genes, differences in their expression between IgAN patients and LDs were examined across three cohorts. The expression levels of the genes *CD160*, *CX3CR1*, *EPHA4*, *THBS1*, *HLA-DRA*, *FARP2*, *VASH1*, *TMSB4Y*, and *RHBDD3* were assessed. In the GSE37460 cohort, genes such as *HLA-DRA*, *VASH1* and *RHBDD3* were significantly more highly expressed in IgAN patients than in LDs patients (all *p* < 0.05), while *THBS1*, *FARP2* and *TMSB4Y* were more highly expressed in LDs patients (Figure 5A). In the GSE93798 cohort, similar patterns were observed; the genes whose expression increased in IgAN patients were not only *HLA-DRA*, *VASH1* and *RHBDD3* but also *CD160* and *CX3CR1*, while high levels of *EPHA4* and *THBS1* were detected in the LDs group (Figure 5B). Finally, in GSE115857, the trends continued, with *HLA-DRA*, *VASH1* and *RHBDD3* markedly higher in IgAN patients, and *FARP2* also showed higher expression levels in the LDs group (Figure 5C). These findings suggest that *HLA-DRA*, *VASH1* and *RHBDD3* play significant roles in distinguishing IgAN from LDs and could serve as potential biomarkers for the diagnosis of IgAN.

**Figure 6.**
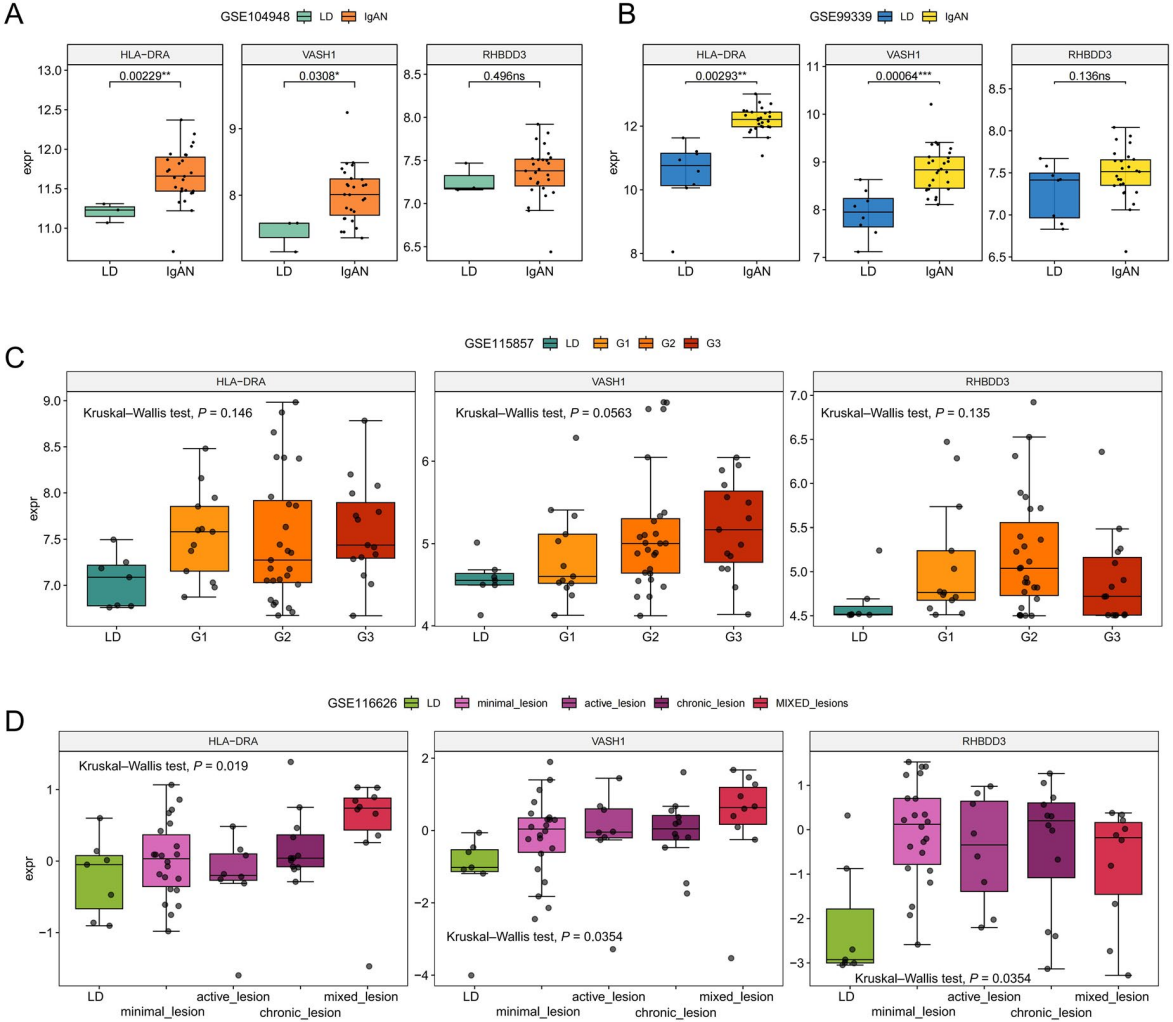
Verification of *HLA-DRA*, *VASH1*, and *RHBDD3* gene expression in external cohorts. (A-B) Box plots showing expression levels in IgAN patients versus living donors in the GSE104948 (A) and GSE99339 (B) cohorts. (C) Box plot showing that the expression trends of *HLA-DRA*, *VASH1* and *RHBDD3* correlate with IgAN severity grade. (D) Box plot showing the correlation of the expression trends of *HLA-DRA*, *VASH1* and *RHBDD3* with IgAN histological subtypes.

We further validated the expression of *HLA-DRA*, *VASH1* and *RHBDD3* in the external cohort. We first observed an increasing trend in the expression of these three genes, especially *HLA-DRA* and *VASH1*, in both the GSE104948 (Figure 6A) and GSE99339 (Figure 6B) cohorts. In the GSE115857 cohort, the IgAN patients were further classified into three severity groups, namely, mild G1, moderate G2 and severe G3, according to the Schena classification. We observed an increasing trend in *HLA-DRA* and *VASH1* with increasing severity (Figure 6C). In the GSE116626 cohort, the IgAN patients were separated into minimal lesion, active lesion, chronic lesion and mixed lesion subtypes. We observed the highest levels of *HLA-DRA* and *VASH1* in mixed lesion IgAN patients, indicating that the increase in these two genes is positively correlated with the pathological severity of IgAN (Figure 6D).

### 3.5. Relationships between key biomarkers and the infiltration of immune cells

**Figure 7.**
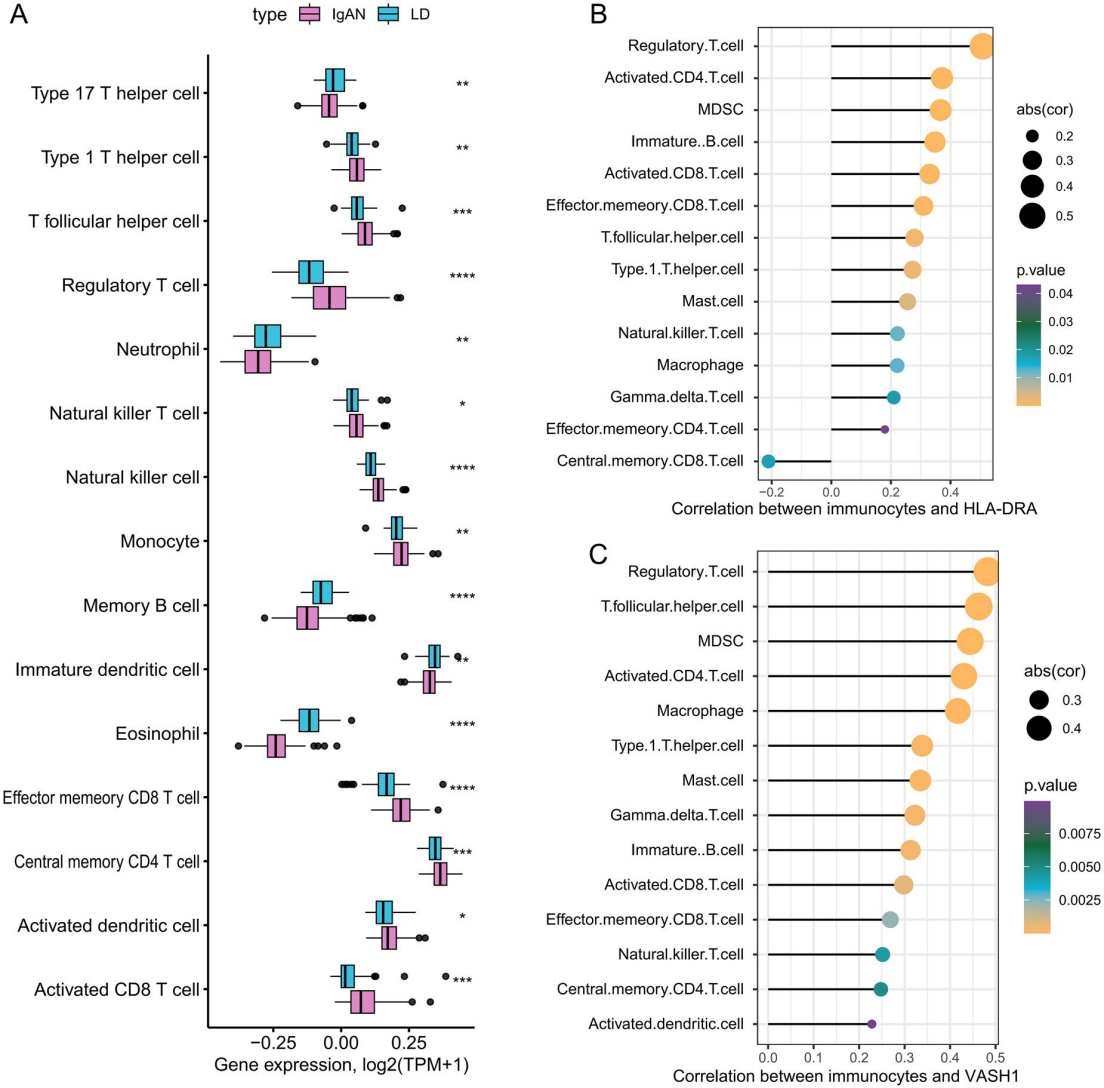
Immune cell discrepancies and gene correlations in IgAN patients. (A) Differential immune cell expression between IgAN patients and LD patients. (B) Correlation of immune cells with *HLA-DRA*. (C) Correlation of immune cells with *VASH1.*

Accumulating data indicate that disruptions in immune system regulation are central to the etiology of IgAN. A discriminative analysis contrasting IgAN patients with healthy individuals was conducted to delineate disparities in immune cell composition. Notably, in the IgAN cohort, there was substantial variation in the expression of several immune cells, especially various subsets of CD8+ T cells, CD4+ T cells, and regulatory T cells (Figure 7A). Subsequent investigations probed the link between the *HLA-DRA* gene and the degree of immune cell infiltration in IgAN patients. The *HLA-DRA* gene was found to correlate differentially with diverse immune cells, exhibiting the most pronounced positive correlations with regulatory T cells, activated CD4+ T cells, and myeloid-derived suppressor cells (MDSCs), as depicted in Figure 7B. Furthermore, *VASH1* was positively correlated with an array of immune cells, with the most substantial links observed with regulatory T cells, T follicular helper cells, and MDSCs (Figure 7C). These findings underscore a noteworthy synergy between the expression profiles of *HLA-DRA* and *VASH1* and the incursion of particular immune cells within the context of IgAN.

**Figure 8.**
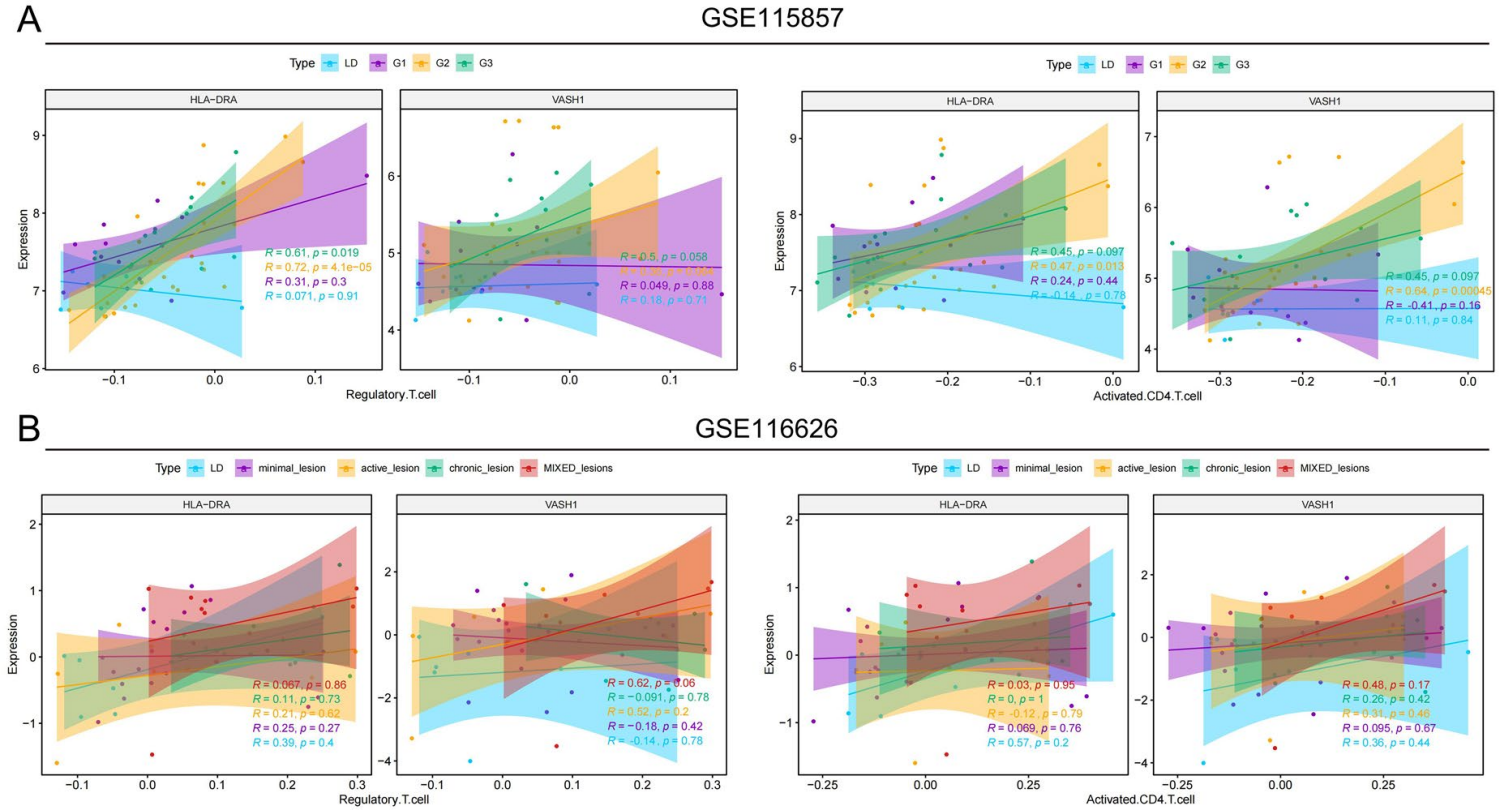
Correlation analysis of *HLA-DRA* and *VASH1* with immunocytes across IgAN subtypes. (A) Scatter plots for GSE115857 showing the correlation between genes and immunocytes across IgAN severity grades. (B) Scatter plots for GSE116626 showing correlations between genes and immunocytes across different IgAN lesion subtypes.

Observations revealed a marked positive correlation between *HLA-DRA*, *VASH1*, and regulatory T cells as well as between HLA-DRA and activated CD4+ T cells. Consequently, a more granular analysis was performed to elucidate the associations of these genes with varying stages of IgAN. Within the GSE115857 cohort, the most significant correlations with regulatory T cells were detected in patients with G3 stage IgAN (*HLA-DRA: R* = 0.61, *p* = 0.019; *VASH1: R* = 0.5, *p* = 0.058; Figure 8A). Additionally, in G2 stage IgAN patients, notable correlations with activated CD4+ T cells were detected (*HLA-DRA: R* = 0.47, *p* = 0.013; *VASH1: R* = 0.64, *p* < 0.01; depicted in Figure 8A). In the GSE116626 cohort, *VASH1* exhibited positive but non-significant trend with regulatory T cells in IgAN patients with mixed lesions (*R* = 0.62, *p* = 0.06), and a similar trend was observed for activated CD4+ T cells (*R* = 0.48, *p* = 0.17, Figure 8B).

### 3.6. Validation of key biomarkers in IgAN

**Figure 9.**
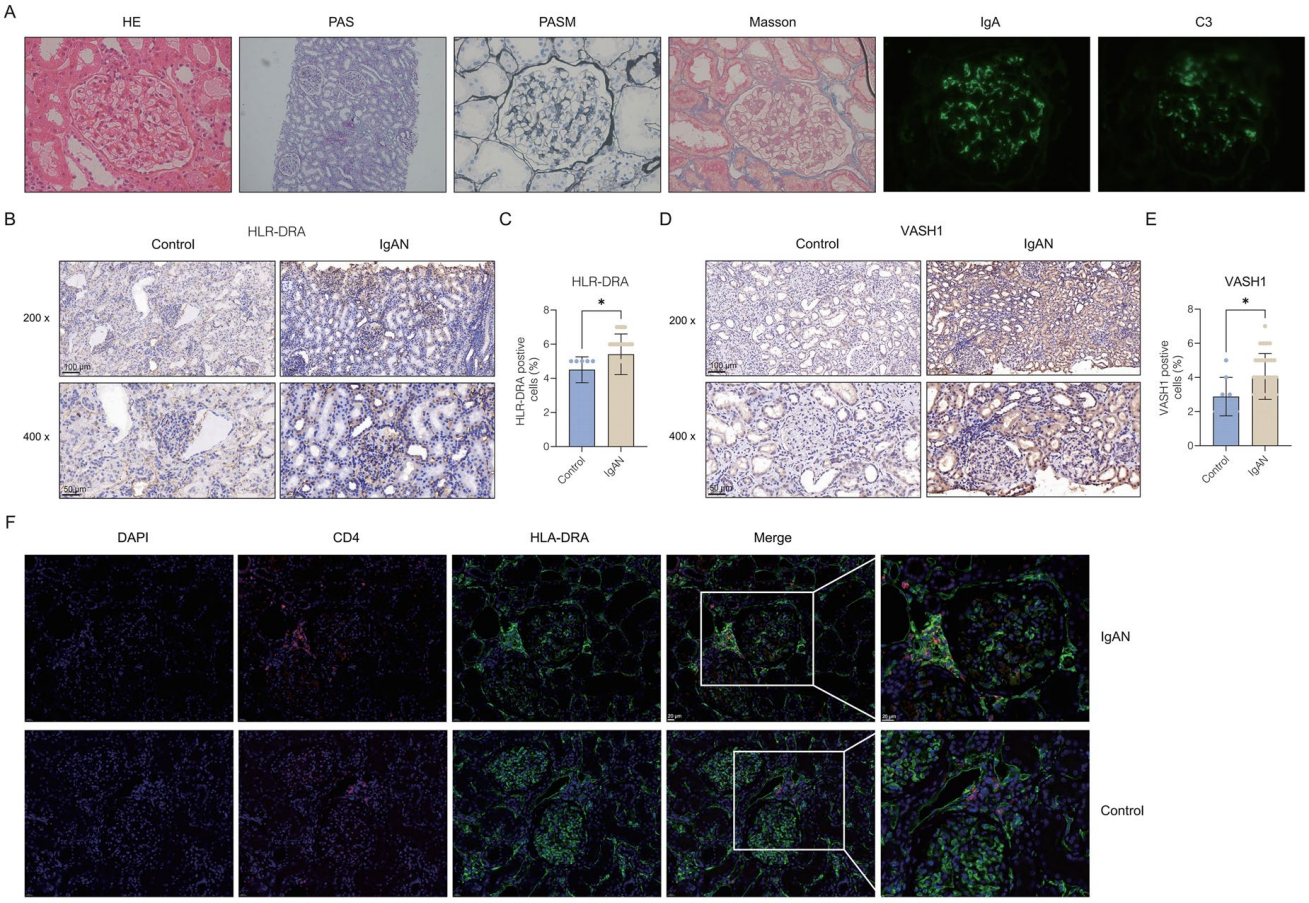
Immunohistochemistry (IHC) and immunofluorescence validation of HLA-DRA and VASH1 as biomarkers in kidney biopsy tissue from patients with IgA nephropathy (IgAN). (A) Pathological diagnostic results of clinical IgAN patients, demonstrating structural and immunological alterations in kidney tissues using various staining techniques: HE, PAS, silver, Masson, IgA, and C3. (B-E) Immunohistochemical validation of HLA-DRA and VASH1 expression in IgAN patients and controls. (B, D) Representative images of immunohistochemical staining for HLA-DRA and VASH1 in IgAN patients and controls. (C, E) Quantitative analysis showing a significant increase in HLA-DRA and VASH1 expression in IgAN patients compared to controls (Mann-Whitney U test, *p < 0.05). (F) Immunofluorescence staining of CD4 and HLA-DRA in kidney tissues, indicating a greater presence of CD4 + HLA-DRA+ functionally active/activated CD4+ T cells in IgAN tissues than in control tissues.

To validate the significance of the markers revealed above, we conducted a comprehensive analysis of patients with IgAN (Table 2). Pathological diagnostic results from clinical patients revealed substantial structural and immunological alterations in kidney tissues using various staining techniques (Figure 9A). IHC validation analysis revealed a pronounced increase in HLA-DRA and VASH1 expression in IgAN patients compared with controls, with semiquantitative scoring analysis confirming statistically significant increases (Mann-Whitney U test, *p* < 0.05; Figure 9B-E). The IHC validation results indicated that HLA-DRA expression was more specific to the glomeruli, with relatively weaker expression in the tubules, whereas VASH1 expression was observed in both glomeruli and tubules in IgAN patients. Additionally, immunofluorescence staining indicated a greater presence of CD4 + HLA-DRA+ functionally active/activated CD4+ T cells in IgAN tissues than in control tissues (Figure 9F). These findings collectively underscore the pathological changes and immune cell infiltration characteristics of IgAN, identifying HLA-DRA and VASH1 as critical biomarkers and highlighting the pivotal role of activated T cells in the disease mechanism.

### 3.7. External calibration and SHAP explainability of a 9-gene IgAN model

**Figure 10.**
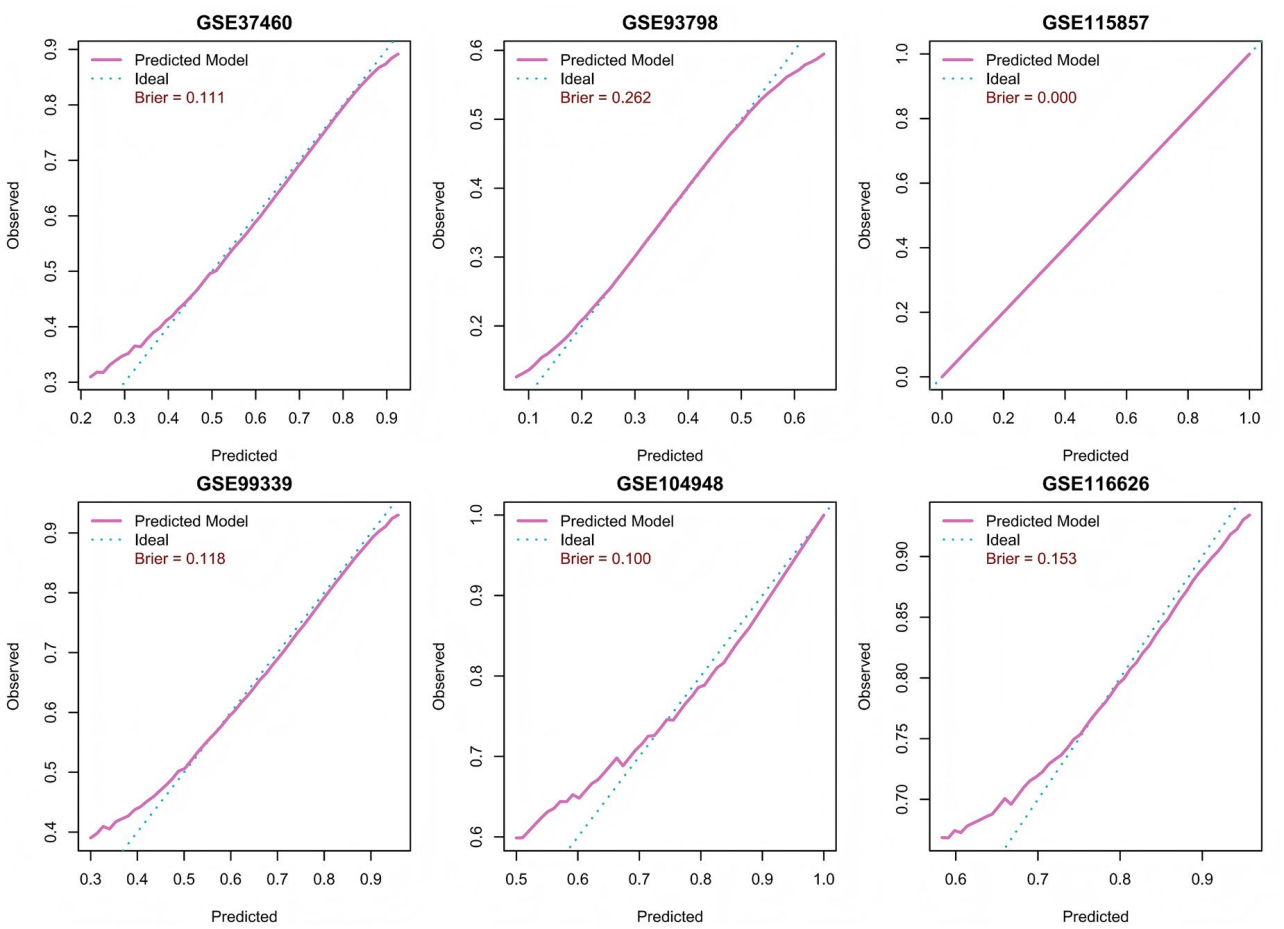
Calibration and Brier scores of the 9-gene model across cohorts. Calibration plots compare predicted probabilities with observed event frequencies for five independent validation cohorts—internal: GSE37460, GSE93798; external: GSE99339, GSE116626, GSE104948. The pink curve shows the model’s predicted calibration; the blue dotted line is the ideal reference (perfect calibration). Closer overlap indicates better agreement and more reliable probability estimates across datasets. The GSE115857 panel is shown for completeness as the training cohort and is not used to claim external performance. Each panel reports the Brier score, which measures the mean squared difference between predicted probabilities and observed outcomes (lower values indicate better overall calibration and accuracy). In our study, the overall Brier score was 0.118, and most cohorts had Brier values < 0.15, supporting good calibration of predicted probabilities across datasets. Axes: x, predicted probability; y, observed frequency.

**Figure 11.**
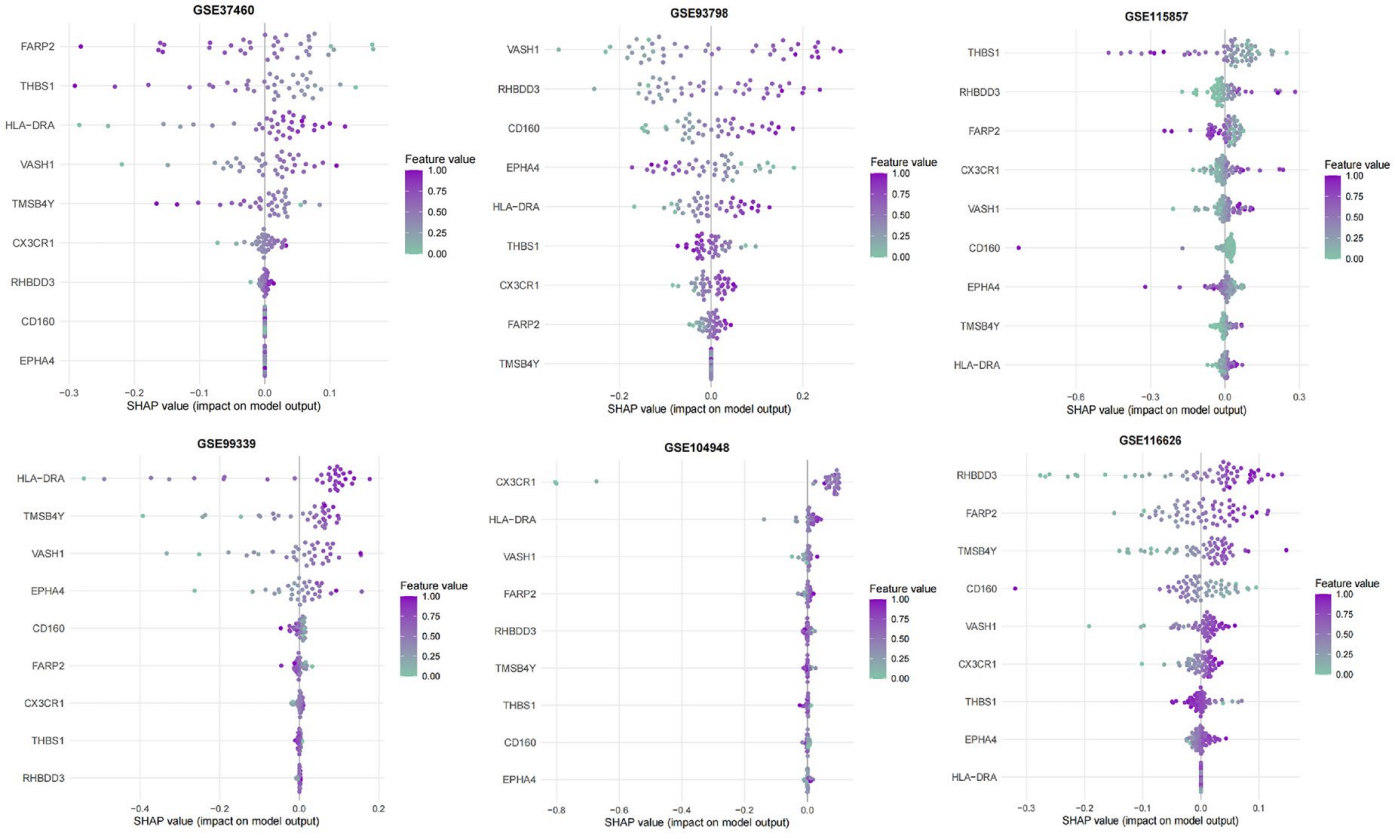
SHAP Explanation of the 9-Gene Diagnostic Model Across Multiple IgAN Cohorts. Summary SHAP (SHapley Additive exPlanations) plot displaying the feature importance of nine genes (CD160, CX3CR1, EPHA4, THBS1, HLA-DRA, FARP2, VASH1, TMSB4Y, RHBDD3) in the machine learning model across six independent cohorts (GSE115857, GSE37460, GSE93798, GSE99339, GSE104948, GSE116626). Each dot represents an individual sample. The x-axis reflects the SHAP value (effect on model output), and dot color corresponds to gene expression level (red = high, blue = low). Genes are ordered by mean absolute SHAP values, highlighting their relative contribution to diagnosis prediction consistently across cohorts. HLA-DRA, VASH1, and RHBDD3 show the highest impact across datasets, supporting their robustness and biological relevance in IgAN pathogenesis.

We added a comprehensive calibration assessment and model explainability analyses. Calibration plots (Figure 10) across five independent validation cohorts (GSE37460, GSE93798, GSE99339, GSE104948, GSE116626) show the predicted curves closely aligned with the ideal reference, indicating reliable probability estimates; calibration was not applied to the training cohort (GSE115857). In our study, the overall Brier score was 0.118, and most cohorts had Brier values < 0.15, supporting good calibration of predicted probabilities across datasets. To enhance interpretability, we performed SHAP(SHapley Additive exPlanations) analyses on an elastic net model (α = 0.4) trained on the nine selected genes (*CD160*, *CX3CR1*, *EPHA4*, *THBS1*, *HLA-DRA*, *FARP2*, *VASH1*, *TMSB4Y*, *RHBDD3*) using data pooled from six cohorts, quantifying per-gene contributions to model output probabilities (Figure 11) and reinforcing the biological plausibility of the features in IgAN pathogenesis.

## 4. Discussion

IgA nephropathy (IgAN) is the most common form of primary glomerulonephritis worldwide, yet its etiology and pathogenesis remain incompletely defined, underscoring the need for reliable noninvasive biomarkers to support diagnosis and risk stratification. Aligned with the four-hit model, several fluid-phase candidates have gained attention: anti-Gd-IgA1 IgG shows favorable performance in some cohorts (sensitivity 89%, specificity 92%), though inter-study heterogeneity persists [18]. A meta-analysis of 762 patients identified 23 serum and urinary markers, with urinary interleukin-6 (IL-6) and transforming growth factor-β1 (TGF-β1) demonstrating the strongest discrimination between patients and healthy controls [19,20]. Complement activation appears central to disease biology, as glomerular C3 deposition correlates with crescents and interstitial fibrosis [21]. In the inflammation-immune activation axis, urinary soluble CD163 associates with corticosteroid responsiveness and renal outcomes [22,23]. Additionally, circulating microRNAs, particularly miR-148 and let-7b, are inversely correlated with estimated glomerular filtration rate (eGFR) and may inform long-term prognosis [24].

Artificial intelligence(AI) is reshaping medical research and care. Following TRIPOD AI improves model reproducibility, verification, and clinical safety [25]. Multi-omics integration, combining genomics, transcriptomics, and proteomics in an end to end analysis, supports precision diagnosis, subtyping, and treatment prediction [26]. In nephrology, digital kidney pathology offers a quantitative, reproducible foundation for AI-augmented diagnosis and risk assessment and enables multicenter standardization [27]. Embedding AI-driven AKI prediction models into the European Heart Surgery Risk Assessment system enables real-time risk identification and timely intervention, enhances perioperative management, and improves patient outcomes [28]. Across 41 studies reviewed by Sabanayagam and colleagues, AI improved screening, risk stratification, outcome prediction, and therapy management in CKD [29]. In IgAN, an AI based urinary proteomics study with 50 patients and matched controls reported over 97% diagnostic sensitivity and accuracy, highlighting the promise of AI driven multi-omics for noninvasive diagnosis and monitoring [30].

Machine learning (ML) has emerged as a transformative force in the field of disease diagnosis, marking a significant shift from traditional diagnostic methods to advanced ML-based models. The integration of ML into diagnostic processes is underpinned by the availability of extensive sequencing data and large-scale biomedical datasets [31,32]. The integration of machine learning techniques with transcriptomics analyses has allowed us to identify key signaling pathways and genes that may contribute to the pathobiology of IgAN, providing new avenues for diagnosis and treatment. The identification of 21 signaling pathways significantly activated in IgAN patients suggested a strong immune and inflammatory component in the disease mechanism, aligning with previous research indicating that immune dysregulation is a hallmark of IgAN [33]. The role of these pathways in modulating the immune response, cellular architecture, metabolism, and neurodevelopment underscores the multifaceted nature of IgAN and its systemic impact beyond the kidneys. The development of a diagnostic model based on machine learning represents a significant advance in our diagnostic capabilities for IgAN. The glmBoost + Enet model, with a select set of 9 genes, exhibited high precision and robustness, as evidenced by strong AUC values and kappa statistics across multiple validation cohorts. This model stands out not only for its predictive accuracy but also for its clinical feasibility, given the small number of genes required for the diagnosis. Our model provides a reproducible, externally validated, and parsimonious 9-gene panel that could serve as a reference standard in future IgAN diagnostic biomarker studies.

Our model is a parsimonious, pathway anchored nine gene expression panel primarily intended for diagnosis, with planned extensions to severity and prognosis. It differs from broad transcriptomic patterns [34–36] that rely on large multigene signatures with limited external validation. Proteomic and urinary biomarkers [37–39] including Gd IgA1, anti Gd IgA1, complement components, urinary proteomics, and exosomal miRNAs offer attractive noninvasive readouts but are typically single analyte or limited panel and may not capture pathway level context. In the present study, we identified HLA-DRA and VASH1 as promising biomarkers for the noninvasive diagnosis of IgAN. VASH1 is quantifiable in plasma and urine by ELISA, and circulating/urinary levels have been associated with renal outcomes and other disease states, supporting feasibility for noninvasive monitoring [40,41]. For HLA-DRA, whole-blood or CD14^+^ monocyte mRNA can be measured by qRT-PCR as a surrogate of antigen-presentation capacity, and reduced expression has been prognostic in systemic infections [42]; moreover, plasma cell-free RNA and urinary extracellular vesicles provide established platforms on which HLA-class II transcripts/proteins (including HLA-DR epitopes) can be detected [43]. The International IgAN Prediction Tool [44] is prognostic (clinical plus pathology) rather than diagnostic; we position our panel as complementary for prebiopsy triage and diagnosis, with downstream integration for risk stratification. Finally, we outline a multicenter, large scale clinical validation to translate this nine gene panel into a noninvasive diagnostic model using blood or urine assays.

Using GEO data, we observed in GSE116626 that HLA-DRA and VASH1 increase with chronic lesion severity, supporting biological plausibility; however, we currently lack longitudinal outcomes to demonstrate associations with risk, prognosis. We will pursue prospective, multicenter studies with larger datasets to link model probabilities and gene signals to eGFR slope, kidney failure, remission.

**Figure 12.**
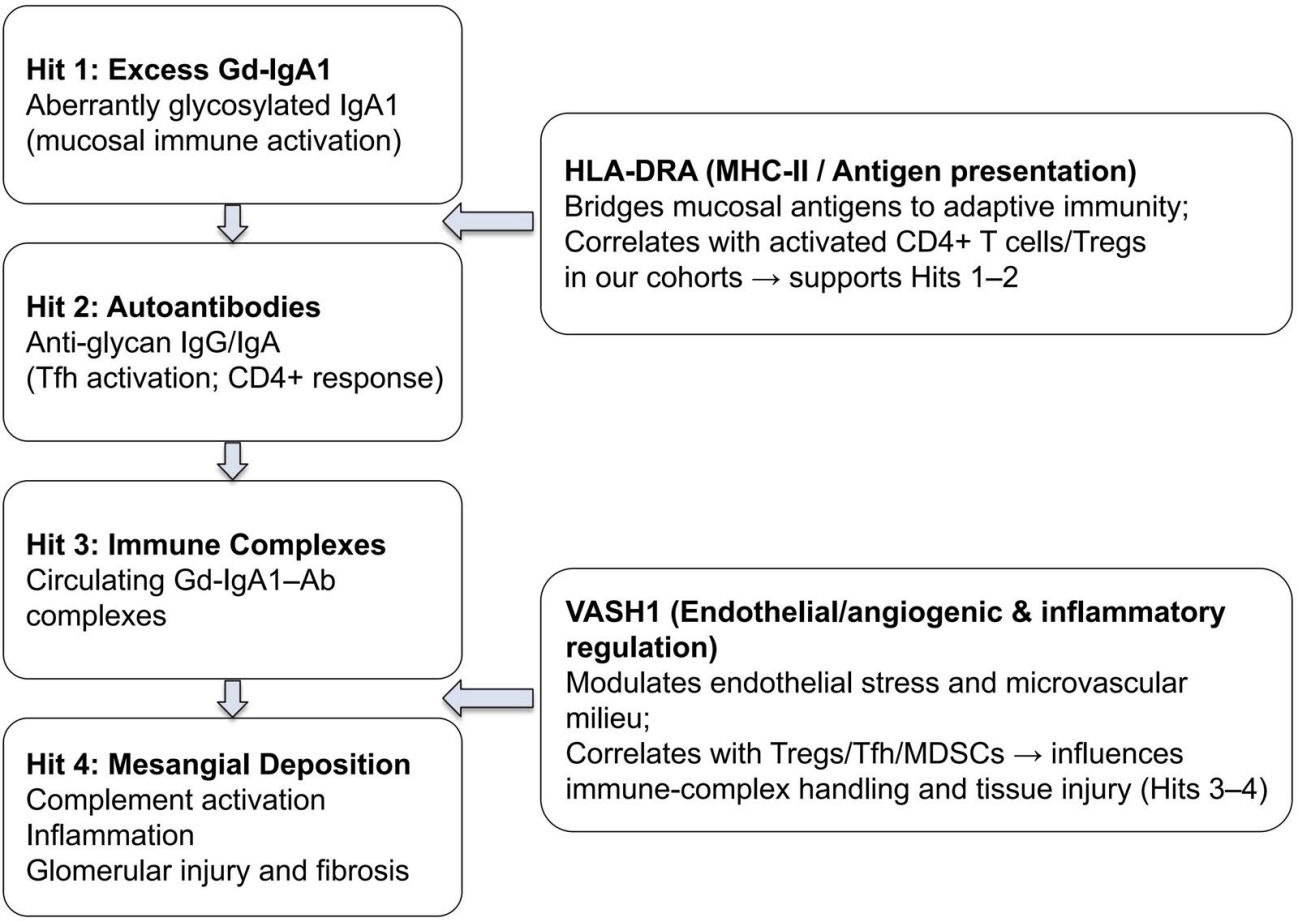
Linking HLA-DRA and VASH1 to the IgAN ‘multi-hit’ hypothesis. Excess galactose-deficient IgA1 triggers anti-Gd-IgA1 autoantibody formation, generating circulating immune complexes that deposit in the mesangium. HLA-DRA localizes to the antigen-presentation axis (hits 1–2: aberrant IgA1 and autoantibody generation), while VASH1 marks endothelial/angiogenic dysregulation (hits 3–4: complex-mediated injury and microvascular remodeling), linking immune activation to mesangial deposition and downstream inflammation.

The genes within the model, such as *HLA-DRA* and *VASH1*, are potentially critical biomarkers for IgAN. *HLA-DRA* forms the α-chain of MHC class II, is expressed on professional antigen-presenting cells, and presents exogenous peptides to CD4^+^ T cells, initiating clonal activation and cytokine production. Beyond kidney disease, altered HLA-DRA expression marks immune surveillance status in cancer and immune suppression in sepsis and transplant settings, underscoring its relevance to broader immunology (Figure 12). HLA-DRA also plays a critical role in the pathogenesis of various diseases. Studies have shown that *HLA-DRA* gene polymorphisms are significantly associated with autoimmune thyroid diseases, such as Graves’ disease and Hashimoto’s thyroiditis [45]. In lupus nephritis (LN), HLA-DRA is significantly expressed in various immune cells and is associated with a low glomerular filtration rate, indicating its important role in the pathology of LN [46]. Additionally, HLA-DRA expression is significantly lower in patients with severe COVID-19 than in mild patients, highlighting its crucial role in modulating the immune response during viral infections [42]. In non-small cell lung cancer, HLA-DRA is associated with an inflamed tumor microenvironment and increased tumor-infiltrating immune cells, suggesting its role in regulating the tumor immune response and treatment efficacy [47]. Although several studies have reported that MHC class II HLA alleles are linked to IgAN [48,49], few studies have focused on their expression. In the context of IgAN, increased expression of HLA-DRA has been linked to the activation and proliferation of T cells, highlights the significant role of the immune system in disease pathology [50]. Besides, the association between HLA-DRA and CD4+ T cell marker co-localization, suggests possible immunoregulatory dysfunction in IgAN, which could be pivotal in both disease progression and intervention.

For the *VASH1* gene, which encodes the protein Vasohibin-1, an endogenous antiangiogenic factor that inhibits angiogenesis and is closely associated with various physiological processes, such as vascular development, tumor growth, tissue repair, and fibrosis [51]. In chronic kidney disease, VASH1 plays a role in regulating angiogenesis and anti-fibrosis functions [52]. Overexpression of VASH1 in acute kidney injury (AKI) and other stress conditions helps maintain endothelial integrity and promote angiogenesis (Figure 12), thereby protecting against vascular injury [53]. However, research on the role of VASH1 in IgA nephropathy is relatively rare.

To validate the significance of the markers revealed above, We further validated these two biomarkers in IgA nephropathy and adjacent cancer tissues. IHC demonstrated markedly increased expression in HLA-DRA and VASH1 expression in IgAN patients, and IF validation indicated a greater presence of CD4 + HLA-DRA+ functionally active/activated CD4+ T cells in IgAN tissues than in controls. These findings collectively underscore the pathological changes and immune cell infiltration characteristics of IgAN, identifying HLA-DRA and VASH1 as critical biomarkers and highlighting the pivotal role of activated T cells in the disease mechanism (Figure 12). However, the roles of these biomarkers in the disease still need to be validated in experimental studies.

This work is among the few studies that integrate multi-cohort machine learning with transcriptomic and histopathologic validation in IgAN. Several limitations should be noted. The small IHC/IF sample size may affect the robustness of the results. Possible control selection bias and the reliance on retrospective GEO datasets with platform and preanalytic heterogeneity may also introduce uncertainty. Moreover, the absence of prospective multicenter validation, especially with noninvasive blood and urine testing, warrants further investigation. Accordingly, our conclusions require confirmation in larger prospective cohorts. We are initiating multicenter studies to assess generalizability, calibration, and clinical utility, and to develop fluid phase readouts such as soluble proteins and cellular or extracellular vesicle RNA aligned with the nine gene panel, particularly HLA-DRA and VASH1, with threshold recalibration against standard measures such as proteinuria, eGFR, and IgA/C3.

Future research should focus on translating the nine-gene diagnostic model into clinically deployable tools through multi-omic integration and prospective validation. Combining transcriptomic, proteomic, and urinary biomarker data with digital pathology could enable noninvasive detection of IgA nephropathy and earlier intervention. Artificial intelligence–driven diagnostic platforms integrating this gene panel within electronic health record systems may further allow automated screening of at-risk populations. In parallel, single-cell and spatial transcriptomic studies are needed to define the cellular origin and spatial dynamics of these nine genes within the glomerulus, providing mechanistic insight into disease progression. Finally, multicenter and longitudinal validation across ethnically diverse cohorts, supported by federated learning frameworks, will be essential to establish generalizability, accelerate clinical translation, and guide precision medicine approaches for IgA nephropathy [54].

## 5. Conclusion

Our multi cohort gene expression and machine learning framework exemplifies precision nephrology by linking molecular signals to actionable diagnosis. IHC confirms differential expression of HLA-DRA and VASH1 in IgA nephropathy, supporting their diagnostic potential. Overall, our reproducible and externally validated nine gene panel could serve as a reference standard for future IgAN biomarker studies, with planned multicenter validation to refine generalizability, calibration, and clinical utility.

## Supplementary Material

supplementary Table 1.docx

Supplementary Figure 2.jpg

Supplementary Table 4.docx

supplementary Table 2.docx

supplementary Table 3.docx

Supplementary Table 5.docx

Supplementary Table 6.docx

Supplementary Figure 1.jpg

## Data Availability

The data that support the findings of this study are available in public repositories. The datasets can be accessed by entering the corresponding accession IDs at the following URL:https://www.ncbi.nlm.nih.gov/geo/. The accession IDs are: GSE104948, GSE115857, GSE116626, GSE37460, GSE93798, and GSE99339.
