# Supplementary figures and images for: A nine-gene diagnostic model for IgA nephropathy based on multi-cohort machine learning: integrating gene expression and immunohistochemical validation
Source: Ren Fail. 2026 Mar 9;48(1):2637355. doi: 10.1080/0886022X.2026.2637355 (PMC12978185; doi:10.1080/0886022X.2026.2637355)

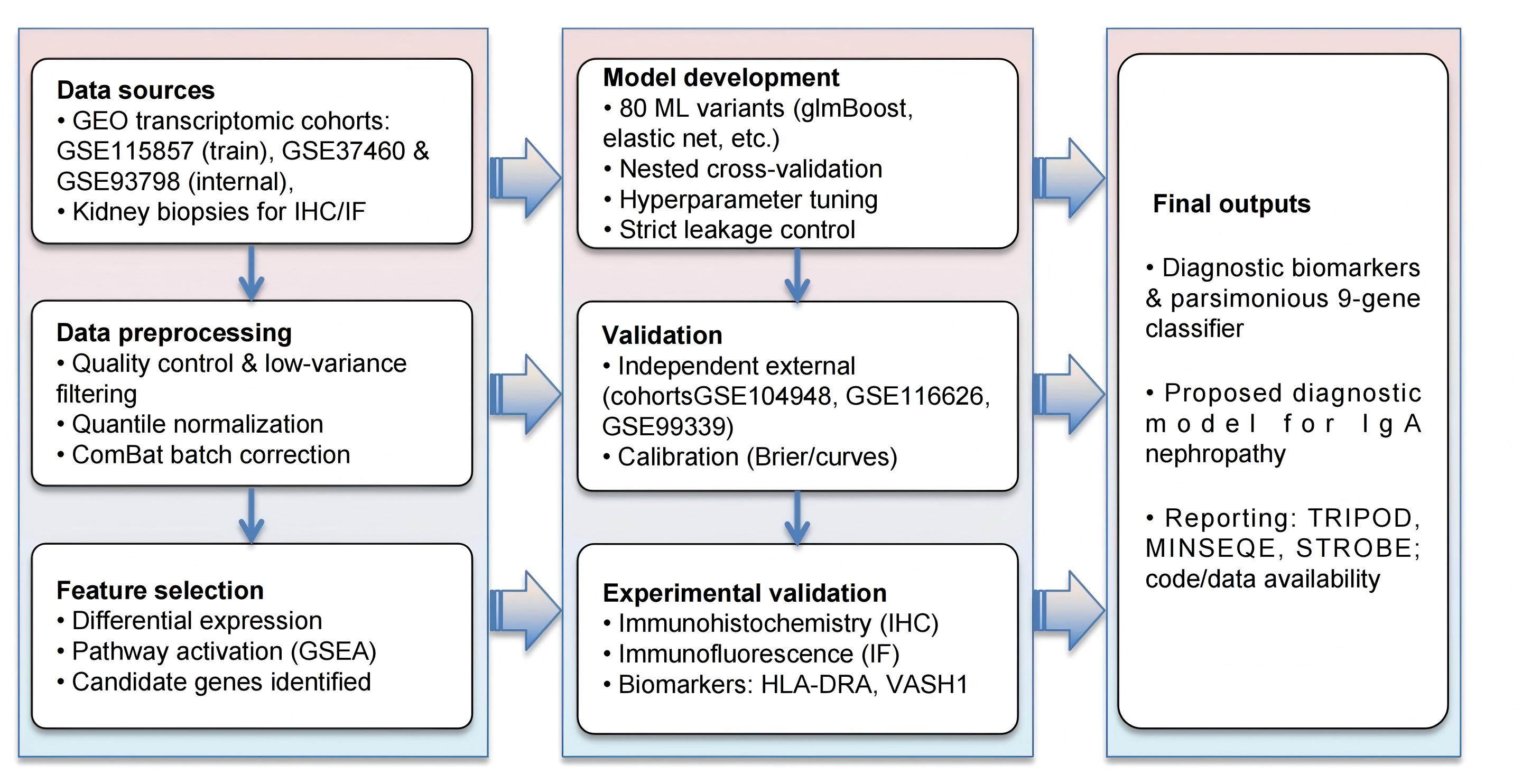

Supplement: Supplementary Figure 2.jpg [file IRNF_A_2637355_SM7523.jpg]

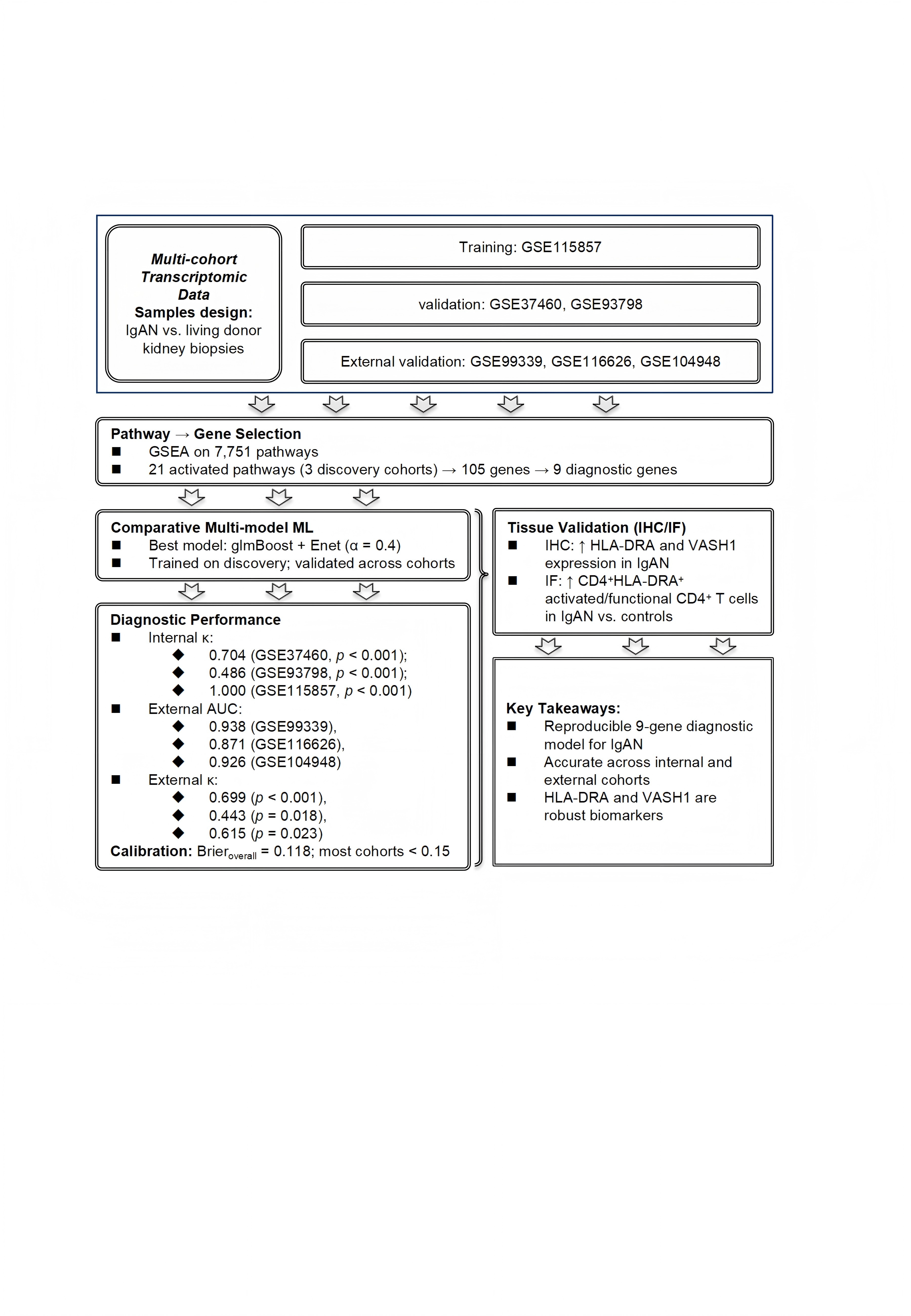

Supplement: Supplementary Figure 1.jpg [file IRNF_A_2637355_SM7516.jpg]
