# Supplementary material for: A nine-gene diagnostic model for IgA nephropathy based on multi-cohort machine learning: integrating gene expression and immunohistochemical validation
Source: Ren Fail. 2026 Mar 9;48(1):2637355. doi: 10.1080/0886022X.2026.2637355 (PMC12978185; doi:10.1080/0886022X.2026.2637355)
Supplement: Supplementary Table 4.docx [file IRNF_A_2637355_SM7522.docx]

**Supplementary Table 4. The 21 distinct signaling pathways and their encompassed genes.**

| Pathway | Genes |
| --- | --- |
| GO_MEMBRANE_RAFT_DISTRIBUTION | DOCK2, GSN, MAL, PTPRC, CD2 |
| GO_NEGATIVE_REGULATION_OF_NATURAL_KILLER_CELL_ACTIVATION | MICA, PIBF1, PGLYRP2, PGLYRP3, CLNK, FGR, RHBDD3, HLA-F, PGLYRP4, HAVCR2, PGLYRP1 |
| GO_MEMBRANE_RAFT_LOCALIZATION | DOCK2, GSN, MAL, PTPRC, RALA, CD2 |
| GO_NEGATIVE_REGULATION_OF_TROPHOBLAST_CELL_MIGRATION | ACVR1C, GJA1, ARHGDIB, MIR15B, NODAL, TIMP1, CALR |
| GO_PEPTIDE_ANTIGEN_ASSEMBLY_WITH_MHC_PROTEIN_COMPLEX | HLA-DMA, HLA-DMB, HLA-DRA, TAPBPL, CALR |
| GO_SEQUESTERING_OF_ACTIN_MONOMERS | TMSB15A, TWF2, TMSB15B, GSN, PFN4, TWF1, TMSB4X, SCIN, TMSB4Y, TMSB10 |
| GO_HEPARIN_METABOLIC_PROCESS | GLCE, ANGPT1, NDST1, IDUA, CSGALNACT1, XYLT2, NDST4, SLC10A7, NDST2, NDST3 |
| GO_REGULATION_OF_LYMPHANGIOGENESIS | CCBE1, EPHA2, VASH1, FOXC1, VEGFC |
| GO_LYMPHOCYTE_AGGREGATION | MSN, JAM2, RAC2, STK10, ZAP70 |
| GO_NEURON_REMODELING | C1QL1, EPHA8, ANKS1A, RND1, APP, BCL11A, ADGRB3, NTN4, CX3CL1, C1QA, C3, SCARF1, FARP2 |
| GO_MHC_PROTEIN_COMPLEX_ASSEMBLY | HLA-DMA, HLA-DMB, HLA-DRA, TAPBPL, TAPBP, CALR |
| GO_COLLAGEN_ACTIVATED_TYROSINE_KINASE_RECEPTOR_SIGNALING_PATHWAY | COL1A1, COL4A1, COL4A2, COL4A3, COL4A4, COL4A5, COL4A6, DDR2, SYK, DDR1, UBASH3B |
| GO_REGULATION_OF_AORTA_MORPHOGENESIS | EFNB2, MIR205, MIR29B1, MIR29B2, NOTCH1 |
| GO_REGULATION_OF_ARTERY_MORPHOGENESIS | EFNB2, MIR153-1, MIR153-2, MIR205, MIR29B1, MIR29B2, MDK, NOTCH1, MIR329-1, MIR329-2, MIR494, MIR495, MIR487B |
| GO_DENDRITIC_CELL_ANTIGEN_PROCESSING_AND_PRESENTATION | NOD1, FGL2, CCR7, FCGR2B, CLEC4A, CCL19, CCL21, NOD2, SLC11A1, THBS1, CD68, CD74 |
| GO_T_CELL_ACTIVATION_VIA_T_CELL_RECEPTOR_CONTACT_WITH_ANTIGEN_BOUND_TO_MHC_MOLECULE_ON_ANTIGEN_PRESENTING_CELL | LILRB1, FGL2, HLA-DMB, ICAM1, ITGAL, LGALS3, LGALS9, APBB1IP, HAVCR2, CD81 |
| GO_SYNAPSE_PRUNING | CDK5, CX3CR1, EPHA4, DKK1, ITGAM, TREM2, CX3CL1, C1QA, C1QB, C1QC, C3 |
| GO_NATURAL_KILLER_CELL_CYTOKINE_PRODUCTION | CD96, CD226, CD160, CLNK, HLA-E, HLA-F, HLA-G, RAET1G, KIR2DL4 |
| GO_GROWTH_INVOLVED_IN_HEART_MORPHOGENESIS | S1PR1, MIR195, NOTCH1, SIRT6, MESP1 |
| GO_REGULATION_OF_COLLAGEN_FIBRIL_ORGANIZATION | EMILIN1, CHADL, AEBP1, MIR29B1, MIR29B2, RB1, COLGALT1 |
| GO_GRANULOCYTE_COLONY_STIMULATING_FACTOR_PRODUCTION | LILRA2, ISL1, HAVCR2, TSLP, CD34 |
