# Supplementary material for: A nine-gene diagnostic model for IgA nephropathy based on multi-cohort machine learning: integrating gene expression and immunohistochemical validation
Source: Ren Fail. 2026 Mar 9;48(1):2637355. doi: 10.1080/0886022X.2026.2637355 (PMC12978185; doi:10.1080/0886022X.2026.2637355)
Supplement: supplementary Table 2.docx [file IRNF_A_2637355_SM7521.docx]

MINSEQE Checklist (Prefilled)-Transcriptomic Profiling

| Item | Requirement / What we report | Manuscript location (section/figure/table) |
| --- | --- | --- |
| Experiment design/metadata | Cohort sources, inclusion/exclusion, clinical metadata overview. | Methods: Basic information; Patient enrollment |
| Platform & annotations | GEO platforms with probe-to-gene mapping; MSigDB C5:BP gene sets (7,751). | Methods: GSEA; Supplementary Table 1 |
| Raw/processed data access | GEO accessions for all cohorts; processed matrices to be provided. | Methods: Basic information; Data availability |
| Preprocessing/QC | Quantile normalization; ComBat batch correction; low-variance filtering implied; strict leakage control. | Methods: Dismissal of Batch Effects; ML training |
| Gene set resources | MSigDB version and categories; ssGSEA gene sets via GSVA. | Methods: GSEA; Infiltration of immune cells |
| Analysis pipeline | Nested CV; randomized/grid search; kNN imputation; pathway anchoring; scripts and environment files to be shared. | Methods: ML training; Predictive model |
| Reproducibility artifacts | Code repository, environment lockfiles, and workflow diagram (Figure S). | Methods: Reporting guidelines; Figure S |
