# Supplementary material for: A nine-gene diagnostic model for IgA nephropathy based on multi-cohort machine learning: integrating gene expression and immunohistochemical validation
Source: Ren Fail. 2026 Mar 9;48(1):2637355. doi: 10.1080/0886022X.2026.2637355 (PMC12978185; doi:10.1080/0886022X.2026.2637355)
Supplement: supplementary Table 3.docx [file IRNF_A_2637355_SM7519.docx]

STROBE Statement—checklist of items that should be included in reports of observational studies

**Item**

**No Recommendation**

| **Title and**  **abstract[1]** | 1 | (*a*) Indicate the study’s design with a commonly used term in the title or the abstract | Title and Abstract include “Multi-Cohort Machine Learning Diagnostic Model” |
| --- | --- | --- | --- |
| (*b*) Provide in the abstract an informative and balanced summary of what was done and what was found | | | Abstract provides objective, methods, results (AUC, Kappa), and conclusions |
| **Introduction** | | | |
| Background/ | 2 | Explain the scientific background and | Introduction Section 1, paragraphs 1–3; discusses |
| rationale |  | rationale for the investigation being reported | IgAN pathogenesis, diagnostic gaps |
| Objectives | 3 | State specific objectives, including any prespecified hypotheses | Introduction final paragraph: to develop and validate a 9-gene diagnostic model for IgAN |
| **Methods** | | | |
| Study design | 4 | Present key elements of study design early in the paper | Methods 2. 1–2.5 describe cohort selection and multi- cohort integration |
| Setting | 5 | Describe the setting, locations, and  relevant dates, including periods of  recruitment, exposure, follow-up, and data collection | Methods 2. 1; GEO datasets GSE115857, GSE37460, GSE93798, GSE99339, GSE116626, GSE104948 |
| Participants | 6 | (*a*) *Cohort study*—Give the eligibility criteria, and the sources and methods of selection of participants. Describe  methods of follow-up  *Case-control study*—Give the eligibility criteria, and the sources and methods of case ascertainment and control selection. Give the rationale for the choice of cases and controls  *Cross-sectional study*—Give the  eligibility criteria, and the sources and methods of selection of participants | Methods 2. 1, 2.6; GEO datasets; IgAN patients confirmed by biopsy, controls from nephrectomy specimens |
|  |  | (*b*) *Cohort study*—For matched studies, give matching criteria and number of  exposed and unexposed  *Case-control study*—For matched studies, give matching criteria and the number of controls per case | Methods 2.6; IgAN patients confirmed by biopsy, controls from nephrectomy specimens |
| Variables | 7 | Clearly define all outcomes, exposures, predictors, potential confounders, and  effect modifiers. Give diagnostic criteria, if applicable | Methods 2.3–2.4; outcomes = IgAN vs control, exposures = gene expression levels |

Data sources/ measurement

8* For each variable of interest, give sources of data and details of methods of

Methods 2.3–2.5; GSEA, ssGSEA, ComBat normalization, R 4.2.2 software

assessment (measurement). Describe

comparability of assessment methods if there is more than one group

| Bias | 9 | Describe any efforts to address potential sources of bias | Methods 2.2; removed batch effects using ComBat; independent validation cohorts |
| --- | --- | --- | --- |
| Study size | 10 | Explain how the study size was arrived at | Methods 2. 1; all available GEO samples (207 IgAN, 56 LDs) included for maximal power |
| Quantitative | 11 | Explain how quantitative variables were | Methods 2.3–2.4; standardized log2-transformed |
| variables |  | handled in the analyses. If applicable, describe which groupings were chosen and why | expression, fold-change cutoff 0.3, p<0.05 |
| Statistical methods | 12 (*a*) Describe all statistical methods, including those used to control for confounding | | Methods 2.9; Fisher’s test, Pearson correlation, t-test, Wilcoxon, Kruskal–Wallis, AUC, Kappa |
|  |  | (*b*) Describe any methods used to  examine subgroups and interactions | Methods 2. 1; Table 1 – numbers per GEO cohort |
|  |  | (*c*) Explain how missing data were addressed | |
|  |  | (*d*) *Cohort study*—If applicable, explain how loss to follow-up was addressed  *Case-control study*—If applicable, explain how matching of cases and controls was addressed  *Cross-sectional study*—If applicable, describe analytical methods taking  account of sampling strategy | |

(*e*) Describe any sensitivity analyses

Continued on next page

Participants 13* (a) Report numbers of individuals at each

stage of study—eg numbers potentially eligible, examined for eligibility,

confirmed eligible, included in the study, completing follow-up, and analysed

Results 3. 1; includes demographic and cohort differences

(b) Give reasons for non-participation at each stage

(c) Consider use of a flow diagram

| Descriptive data  14* | | (a) Give characteristics of study  participants (eg demographic, clinical,  social) and information on exposures and potential confounders | |
| --- | --- | --- | --- |
|  |  | (b) Indicate number of participants with missing data for each variable of interest | |
|  |  | (c) *Cohort study*—Summarise follow-up time (eg, average and total amount) | |
| Outcome data  15* | | *Cohort study*—Report numbers of  outcome events or summary measures over time | |
|  |  | *Case-control study—*Report numbers in each exposure category, or summary  Results 3.2–3.3; AUC and Kappa values for each cohort, summarized in Figures 3–4  measures of exposure | |
|  |  | *Cross-sectional study—*Report numbers of outcome events or summary measures | |
| Main results | 16 | (*a*) Give unadjusted estimates and, if  applicable, confounder-adjusted estimates and their precision (eg, 95% confidence  interval). Make clear which confounders were adjusted for and why they were  included | Results 3.2–3.7; includes cross-cohort validation, SHAP feature contributions |

(*b*) Report category boundaries when

continuous variables were categorized

(*c*) If relevant, consider translating

estimates of relative risk into absolute risk for a meaningful time period

| Other | 17 | Report other analyses done—eg analyses | Results 3.7; SHAP explainability and calibration |
| --- | --- | --- | --- |
| analyses |  | of subgroups and interactions, and sensitivity analyses | across external cohorts |
| **Discussion** |  |  |  |
| Key results | 18 | Summarise key results with reference to study objectives | Discussion, paragraph 1; summarizes 9-gene diagnostic model performance |

Limitations 19 Discuss limitations of the study, taking

into account sources of potential bias or imprecision. Discuss both direction and magnitude of any potential bias

Discussion, last two paragraphs; mentions small IHC/IF sample, retrospective datasets

Interpretatio n

20 Give a cautious overall interpretation of results considering objectives, limitations, multiplicity of analyses, results from

similar studies, and other relevant evidence

Discussion, middle; integrates AI relevance and diagnostic potential of biomarkers

| Generalisabil | 21 | Discuss the generalisability (external | Discussion, final paragraphs; external validation |
| --- | --- | --- | --- |
| ity |  | validity) of the study results | across 6 cohorts, plan for multicenter study |
| **Other information** | | | |

Funding 22 Give the source of funding and the role of

the funders for the present study and, if

applicable, for the original study on which the present article is based

Funding section; “No funds, grants, or other support were received.”

*Give information separately for cases and controls in case-control studies and, if applicable, for exposed and unexposed groups in cohort and cross-sectional studies.

**Note:** An Explanation and Elaboration article discusses each checklist item and gives methodological background and published examples of transparent reporting. The STROBE checklist is best used in conjunction with this article (freely available on the Web sites of PLoS Medicine at <http://www.plosmedicine.org/>, Annals of Internal Medicine at

<http://www.annals.org/>, and Epidemiology at <http://www.epidem.com/>). Information on the STROBE Initiative is available at www.strobe-statement.org.
