# Supplementary material for: A nine-gene diagnostic model for IgA nephropathy based on multi-cohort machine learning: integrating gene expression and immunohistochemical validation
Source: Ren Fail. 2026 Mar 9;48(1):2637355. doi: 10.1080/0886022X.2026.2637355 (PMC12978185; doi:10.1080/0886022X.2026.2637355)
Supplement: Supplementary Table 5.docx [file IRNF_A_2637355_SM7518.docx]

**Supplementary Table 5. The selected genes from the most activated pathways subjected to the 80 algorithmic combinations.**

| Pathway | Genes |
| --- | --- |
| Enet[alpha=0.1] | LILRA2, ISL1, CD34, EMILIN1, AEBP1, RB1, S1PR1, SIRT6, CD96, CD160, HLA_E, HLA_F, CDK5, CX3CR1, EPHA4, ITGAM, TREM2, CX3CL1, C1QA, C1QB, LILRB1, FGL2, HLA-DMB, ICAM1, ITGAL, LGALS9, CD81, NOD1, CCR7, CLEC4A, CCL19, NOD2, THBS1, CD68, CD74, MDK, COL4A1, COL4A2, COL4A3, COL4A4, COL4A5, COL4A6, DDR2, SYK, DDR1, HLA_DMA, HLA_DRA, TAPBPL, TAPBP, CALR, ANKS1A, RND1, APP, BCL11A, SCARF1, FARP2, MSN, JAM2, STK10, ZAP70, EPHA2, VASH1, ANGPT1, IDUA, XYLT2, NDST4, NDST2, NDST3, TMSB15A, TWF2, GSN, SCIN, TMSB4Y, ARHGDIB, TIMP1, DOCK2, MAL, PTPRC, RALA, CD2, MICA, PIBF1, RHBDD3, PGLYRP4, PGLYRP1 |
| Enet[alpha=0.2] | LILRA2, ISL1, CD34, AEBP1, CD160, CDK5, CX3CR1, EPHA4, CX3CL1, CCR7, CCL19, THBS1, COL4A1, COL4A5, SYK, HLA_DRA, TAPBP, CALR, APP, SCARF1, FARP2, VASH1, ANGPT1, IDUA, TMSB15A, GSN, TMSB4Y, TIMP1, MICA, RHBDD3, PGLYRP4, PGLYRP1 |
| Enet[alpha=0.3] | LILRA2, ISL1, CD34, AEBP1, CD160, CDK5, CX3CR1, EPHA4, CCR7, CCL19, THBS1, COL4A1, COL4A5, SYK, HLA_DRA, TAPBP, APP, SCARF1, FARP2, VASH1, ANGPT1, IDUA, TMSB15A, GSN, TMSB4Y, TIMP1, MICA, RHBDD3, PGLYRP4, PGLYRP1 |
| Enet[alpha=0.4] | LILRA2, ISL1, CD34, AEBP1, CD160, CX3CR1, EPHA4, CCR7, THBS1, COL4A1, COL4A5, SYK, HLA_DRA, FARP2, VASH1, IDUA, GSN, TMSB4Y, TIMP1, RHBDD3, PGLYRP1 |
| Enet[alpha=0.5] | LILRA2, ISL1, CD34, AEBP1, CD160, CX3CR1, EPHA4, CCR7, THBS1, COL4A1, COL4A5, SYK, HLA_DRA, FARP2, VASH1, ANGPT1, IDUA, GSN, TMSB4Y, TIMP1, RHBDD3, PGLYRP1 |
| Enet[alpha=0.6] | LILRA2, ISL1, CD34, AEBP1, S1PR1, NOTCH1, SIRT6, CD96, CD160, HLA_E, HLA_F, HLA_G, CDK5, CX3CR1, EPHA4, DKK1, ITGAM, TREM2, CX3CL1, C1QA, C1QB, C3, LILRB1, FGL2, HLA-DMB, ICAM1, ITGAL, LGALS9, APBB1IP, CD81, NOD1, CCR7, FCGR2B, CLEC4A, CCL19, NOD2, SLC11A1, THBS1, CD68, CD74, EFNB2, MDK, COL1A1, COL4A1, COL4A2, COL4A4, COL4A5, COL4A6, DDR2, SYK, DDR1, HLA_DMA, HLA_DRA, TAPBPL, TAPBP, CALR, C1QL1, ANKS1A, RND1, APP, BCL11A, SCARF1, FARP2, MSN, JAM2, STK10, ZAP70, VASH1, VEGFC, GLCE, ANGPT1, NDST1, IDUA, CSGALNACT1, XYLT2, NDST4, NDST3, TMSB15A, GSN, SCIN, TMSB4Y, TMSB10, GJA1, ARHGDIB, TIMP1, MAL, PTPRC, RALA, CD2, MICA, PIBF1, RHBDD3, PGLYRP4, PGLYRP1 |
| Enet[alpha=0.7] | LILRA2, ISL1, CD34, EMILIN1, AEBP1, S1PR1, NOTCH1, SIRT6, CD96, CD160, HLA_E, HLA_F, HLA_G, CDK5, CX3CR1, EPHA4, DKK1, ITGAM, TREM2, CX3CL1, C1QA, C1QB, C3, LILRB1, FGL2, HLA-DMB, ICAM1, ITGAL, LGALS9, APBB1IP, CD81, NOD1, CCR7, FCGR2B, CLEC4A, CCL19, NOD2, SLC11A1, THBS1, CD68, CD74, EFNB2, MDK, COL1A1, COL4A1, COL4A2, COL4A3, COL4A4, COL4A5, COL4A6, DDR2, SYK, DDR1, HLA_DMA, HLA_DRA, TAPBPL, TAPBP, CALR, C1QL1, ANKS1A, RND1, APP, BCL11A, SCARF1, FARP2, MSN, JAM2, STK10, ZAP70, EPHA2, VASH1, VEGFC, GLCE, ANGPT1, NDST1, IDUA, CSGALNACT1, XYLT2, NDST4, NDST2, NDST3, TMSB15A, TWF2, GSN, SCIN, TMSB4Y, TMSB10, GJA1, ARHGDIB, TIMP1, DOCK2, MAL, PTPRC, RALA, CD2, MICA, PIBF1, FGR, RHBDD3, PGLYRP4, PGLYRP1 |
| Enet[alpha=0.9] | LILRA2, ISL1, CD34, AEBP1, S1PR1, CD160, HLA_E, CDK5, CX3CR1, EPHA4, CX3CL1, C3, LILRB1, ICAM1, ITGAL, CCR7, NOD2, SLC11A1, THBS1, COL1A1, COL4A1, COL4A5, COL4A6, SYK, HLA_DRA, CALR, APP, BCL11A, SCARF1, JAM2, EPHA2, GLCE, IDUA, XYLT2, GSN, TMSB4Y, TMSB10, TIMP1, DOCK2, MAL, CD2, MICA, RHBDD3, PGLYRP1 |
| GBM | ISL1, AEBP1, PIBF1, THBS1, RHBDD3, TMSB4Y, VASH1, EPHA4, C1QL1, CD96, DDR1, FARP2, NDST2, RAC2, LILRA2, SYK, PGLYRP4, COL4A1, IDUA, PGLYRP1, TAPBP, CD34, HLA_DRA, ZAP70, FGL2, MICA, CCL19, APP, LILRB1, BCL11A, SCARF1, APBB1IP, TIMP1, NDST1, CDK5, COL4A6, ICAM1, CD160, EPHA2, NDST3, CD2, CSGALNACT1, CX3CL1, NOTCH1, SIRT6, HLA_DMA, MSN, CX3CR1, EFNB2, COL4A2, STK10, NDST4, FCGR2B, FGR, NOD1, DKK1, ANGPT1, GSN, GJA1, VEGFC, COL4A5, TWF2, GLCE, CCL21, COL1A1, ARHGDIB, `HLA-DMB`, C1QA, LGALS9, RB1, EMILIN1, CD226, CLEC4A, ITGAL, CCR7, TAPBPL, CALR, HLA_E, S1PR1, PTPRC, SLC11A1, TMSB15A, MDK, CD68, DOCK2, ANKS1A, C3, NOD2, HLA_G, SCIN, RND1, CD81, COL4A3, HLA_F, COL4A4, ITGAM, JAM2, CD74, TMSB10, RALA, DDR2, XYLT2, C1QB, TREM2, MAL |
| glmBoost | LILRA2, ISL1, CD160, CX3CR1, EPHA4, THBS1, HLA_DRA, FARP2, VASH1, TMSB4Y, RHBDD3 |
| glmBoost+Enet[alpha=0.1] | CD160, CX3CR1, EPHA4, THBS1, HLA_DRA, FARP2, VASH1, TMSB4Y, RHBDD3 |
| glmBoost+Enet[alpha=0.2] | CD160, CX3CR1, EPHA4, THBS1, HLA_DRA, FARP2, VASH1, TMSB4Y, RHBDD3 |
| glmBoost+Enet[alpha=0.3] | CD160, CX3CR1, EPHA4, THBS1, HLA_DRA, FARP2, VASH1, TMSB4Y, RHBDD3 |
| glmBoost+Enet[alpha=0.4] | CD160, CX3CR1, EPHA4, THBS1, HLA_DRA, FARP2, VASH1, TMSB4Y, RHBDD3 |
| glmBoost+Enet[alpha=0.5] | CD160, CX3CR1, EPHA4, THBS1, HLA_DRA, FARP2, VASH1, TMSB4Y, RHBDD3 |
| glmBoost+Enet[alpha=0.6] | CD160, CX3CR1, EPHA4, THBS1, HLA_DRA, FARP2, VASH1, TMSB4Y, RHBDD3 |
| glmBoost+Enet[alpha=0.7] | CD160, CX3CR1, EPHA4, THBS1, HLA_DRA, FARP2, VASH1, TMSB4Y, RHBDD3 |
| glmBoost+Enet[alpha=0.8] | CD160, CX3CR1, EPHA4, THBS1, HLA_DRA, FARP2, VASH1, TMSB4Y, RHBDD3 |
| glmBoost+Enet[alpha=0.9] | CD160, CX3CR1, EPHA4, THBS1, HLA_DRA, FARP2, VASH1, TMSB4Y, RHBDD3 |
| glmBoost+GBM | THBS1, VASH1, EPHA4, RHBDD3, TMSB4Y, FARP2, CD160, HLA_DRA, CX3CR1 |
| glmBoost+Lasso | CD160, CX3CR1, EPHA4, THBS1, HLA_DRA, FARP2, VASH1, TMSB4Y, RHBDD3 |
| glmBoost+LDA | CD160, CX3CR1, EPHA4, THBS1, HLA_DRA, FARP2, VASH1, TMSB4Y, RHBDD3 |
| glmBoost+NaiveBayes | CD160, CX3CR1, EPHA4, THBS1, HLA_DRA, FARP2, VASH1, TMSB4Y, RHBDD3 |
| glmBoost+plsRglm | CD160, CX3CR1, EPHA4, THBS1, HLA_DRA, FARP2, VASH1, TMSB4Y, RHBDD3 |
| glmBoost+Ridge | CD160, CX3CR1, EPHA4, THBS1, HLA_DRA, FARP2, VASH1, TMSB4Y, RHBDD3 |
| glmBoost+Stepglm[backward] | CD160, CX3CR1, THBS1, RHBDD3 |
| glmBoost+Stepglm[both] | CD160, CX3CR1, THBS1, RHBDD3 |
| glmBoost+Stepglm[forward] | CD160, CX3CR1, EPHA4, THBS1, HLA_DRA, FARP2, VASH1, TMSB4Y, RHBDD3 |
| glmBoost+SVM | CD160, CX3CR1, EPHA4, THBS1, HLA_DRA, FARP2, VASH1, TMSB4Y, RHBDD3 |
| Lasso+GBM | ISL1, THBS1, RHBDD3, VASH1, TMSB4Y, EPHA4, FARP2, CD34, HLA_DRA, LILRA2, CX3CR1, CD160 |
| Lasso+glmBoost | LILRA2, ISL1, CD160, CX3CR1, EPHA4, THBS1, HLA_DRA, FARP2, VASH1, TMSB4Y, RHBDD3 |
| Lasso+LDA | LILRA2, ISL1, CD34, CD160, CX3CR1, EPHA4, THBS1, HLA_DRA, FARP2, VASH1, TMSB4Y, RHBDD3 |
| Lasso+NaiveBayes | LILRA2, ISL1, CD34, CD160, CX3CR1, EPHA4, THBS1, HLA_DRA, FARP2, VASH1, TMSB4Y, RHBDD3 |
| Lasso+plsRglm | LILRA2, ISL1, CD34, CD160, CX3CR1, EPHA4, THBS1, HLA_DRA, FARP2, VASH1, TMSB4Y, RHBDD3 |
| Lasso+Stepglm[backward] | CD160, EPHA4, THBS1, VASH1 |
| Lasso+Stepglm[both] | CD160, EPHA4, THBS1, VASH1 |
| Lasso+Stepglm[forward] | LILRA2, ISL1, CD34, CD160, CX3CR1, EPHA4, THBS1, HLA_DRA, FARP2, VASH1, TMSB4Y, RHBDD3 |
| Lasso+SVM | LILRA2, ISL1, CD34, CD160, CX3CR1, EPHA4, THBS1, HLA_DRA, FARP2, VASH1, TMSB4Y, RHBDD3 |
| LDA | LILRA2, ISL1, CD34, EMILIN1, AEBP1, RB1, S1PR1, NOTCH1, SIRT6, CD96, CD226, CD160, HLA_E, HLA_F, HLA_G, CDK5, CX3CR1, EPHA4, DKK1, ITGAM, TREM2, CX3CL1, C1QA, C1QB, C3, LILRB1, FGL2, `HLA-DMB`, ICAM1, ITGAL, LGALS9, APBB1IP, CD81, NOD1, CCR7, FCGR2B, CLEC4A, CCL19, CCL21, NOD2, SLC11A1, THBS1, CD68, CD74, EFNB2, MDK, COL1A1, COL4A1, COL4A2, COL4A3, COL4A4, COL4A5, COL4A6, DDR2, SYK, DDR1, HLA_DMA, HLA_DRA, TAPBPL, TAPBP, CALR, C1QL1, ANKS1A, RND1, APP, BCL11A, SCARF1, FARP2, MSN, JAM2, RAC2, STK10, ZAP70, EPHA2, VASH1, VEGFC, GLCE, ANGPT1, NDST1, IDUA, CSGALNACT1, XYLT2, NDST4, NDST2, NDST3, TMSB15A, TWF2, GSN, SCIN, TMSB4Y, TMSB10, GJA1, ARHGDIB, TIMP1, DOCK2, MAL, PTPRC, RALA, CD2, MICA, PIBF1, FGR, RHBDD3, PGLYRP4, PGLYRP1 |
| NaiveBayes | LILRA2, ISL1, CD34, EMILIN1, AEBP1, RB1, S1PR1, NOTCH1, SIRT6, CD96, CD226, CD160, HLA_E, HLA_F, HLA_G, CDK5, CX3CR1, EPHA4, DKK1, ITGAM, TREM2, CX3CL1, C1QA, C1QB, C3, LILRB1, FGL2, HLA-DMB, ICAM1, ITGAL, LGALS9, APBB1IP, CD81, NOD1, CCR7, FCGR2B, CLEC4A, CCL19, CCL21, NOD2, SLC11A1, THBS1, CD68, CD74, EFNB2, MDK, COL1A1, COL4A1, COL4A2, COL4A3, COL4A4, COL4A5, COL4A6, DDR2, SYK, DDR1, HLA_DMA, HLA_DRA, TAPBPL, TAPBP, CALR, C1QL1, ANKS1A, RND1, APP, BCL11A, SCARF1, FARP2, MSN, JAM2, RAC2, STK10, ZAP70, EPHA2, VASH1, VEGFC, GLCE, ANGPT1, NDST1, IDUA, CSGALNACT1, XYLT2, NDST4, NDST2, NDST3, TMSB15A, TWF2, GSN, SCIN, TMSB4Y, TMSB10, GJA1, ARHGDIB, TIMP1, DOCK2, MAL, PTPRC, RALA, CD2, MICA, PIBF1, FGR, RHBDD3, PGLYRP4, PGLYRP1 |
| plsRglm | LILRA2, ISL1, CD34, EMILIN1, AEBP1, RB1, S1PR1, NOTCH1, SIRT6, CD96, CD226, CD160, HLA_E, HLA_F, HLA_G, CDK5, CX3CR1, EPHA4, DKK1, ITGAM, TREM2, CX3CL1, C1QA, C1QB, C3, LILRB1, FGL2, X.HLA.DMB., ICAM1, ITGAL, LGALS9, APBB1IP, CD81, NOD1, CCR7, FCGR2B, CLEC4A, CCL19, CCL21, NOD2, SLC11A1, THBS1, CD68, CD74, EFNB2, MDK, COL1A1, COL4A1, COL4A2, COL4A3, COL4A4, COL4A5, COL4A6, DDR2, SYK, DDR1, HLA_DMA, HLA_DRA, TAPBPL, TAPBP, CALR, C1QL1, ANKS1A, RND1, APP, BCL11A, SCARF1, FARP2, MSN, JAM2, RAC2, STK10, ZAP70, EPHA2, VASH1, VEGFC, GLCE, ANGPT1, NDST1, IDUA, CSGALNACT1, XYLT2, NDST4, NDST2, NDST3, TMSB15A, TWF2, GSN, SCIN, TMSB4Y, TMSB10, GJA1, ARHGDIB, TIMP1, DOCK2, MAL, PTPRC, RALA, CD2, MICA, PIBF1, FGR, RHBDD3, PGLYRP4, PGLYRP1 |
| Ridge | LILRA2, ISL1, CD34, EMILIN1, AEBP1, RB1, S1PR1, NOTCH1, SIRT6, CD96, CD226, CD160, HLA_E, HLA_F, HLA_G, CDK5, CX3CR1, EPHA4, DKK1, ITGAM, TREM2, CX3CL1, C1QA, C1QB, C3, LILRB1, FGL2, HLA-DMB, ICAM1, ITGAL, LGALS9, APBB1IP, CD81, NOD1, CCR7, FCGR2B, CLEC4A, CCL19, CCL21, NOD2, SLC11A1, THBS1, CD68, CD74, EFNB2, MDK, COL1A1, COL4A1, COL4A2, COL4A3, COL4A4, COL4A5, COL4A6, DDR2, SYK, DDR1, HLA_DMA, HLA_DRA, TAPBPL, TAPBP, CALR, C1QL1, ANKS1A, RND1, APP, BCL11A, SCARF1, FARP2, MSN, JAM2, RAC2, STK10, ZAP70, EPHA2, VASH1, VEGFC, GLCE, ANGPT1, NDST1, IDUA, CSGALNACT1, XYLT2, NDST4, NDST2, NDST3, TMSB15A, TWF2, GSN, SCIN, TMSB4Y, TMSB10, GJA1, ARHGDIB, TIMP1, DOCK2, MAL, PTPRC, RALA, CD2, MICA, PIBF1, FGR, RHBDD3, PGLYRP4, PGLYRP1 |
| Stepglm[backward] | CD34, SIRT6, C3, CCR7, CCL19, THBS1 |
| Stepglm[backward]+Enet[alpha=0.1] | CD34, SIRT6, C3, CCR7, CCL19, THBS1 |
| Stepglm[backward]+Enet[alpha=0.2] | CD34, SIRT6, C3, CCR7, CCL19, THBS1 |
| Stepglm[backward]+Enet[alpha=0.3] | CD34, SIRT6, C3, CCR7, CCL19, THBS1 |
| Stepglm[backward]+Enet[alpha=0.4] | CD34, SIRT6, C3, CCR7, CCL19, THBS1 |
| Stepglm[backward]+Enet[alpha=0.5] | CD34, SIRT6, C3, CCR7, CCL19, THBS1 |
| Stepglm[backward]+Enet[alpha=0.6] | CD34, SIRT6, C3, CCR7, CCL19, THBS1 |
| Stepglm[backward]+Enet[alpha=0.7] | CD34, SIRT6, C3, CCR7, CCL19, THBS1 |
| Stepglm[backward]+Enet[alpha=0.8] | CD34, SIRT6, C3, CCR7, CCL19, THBS1 |
| Stepglm[backward]+Enet[alpha=0.9] | CD34, SIRT6, C3, CCR7, CCL19, THBS1 |
| Stepglm[backward]+GBM | THBS1, CD34, CCL19, C3, SIRT6, CCR7 |
| Stepglm[backward]+glmBoost | CD34, SIRT6, C3, CCR7, CCL19, THBS1 |
| Stepglm[backward]+Lasso | CD34, SIRT6, C3, CCR7, CCL19, THBS1 |
| Stepglm[backward]+LDA | CD34, SIRT6, C3, CCR7, CCL19, THBS1 |
| Stepglm[backward]+NaiveBayes | CD34, SIRT6, C3, CCR7, CCL19, THBS1 |
| Stepglm[backward]+plsRglm | CD34, SIRT6, C3, CCR7, CCL19, THBS1 |
| Stepglm[backward]+Ridge | CD34, SIRT6, C3, CCR7, CCL19, THBS1 |
| Stepglm[backward]+SVM | CD34, SIRT6, C3, CCR7, CCL19, THBS1 |
| Stepglm[both] | CD34, SIRT6, C3, CCR7, CCL19, THBS1 |
| Stepglm[both]+Enet[alpha=0.1] | CD34, SIRT6, C3, CCR7, CCL19, THBS1 |
| Stepglm[both]+Enet[alpha=0.2] | CD34, SIRT6, C3, CCR7, CCL19, THBS1 |
| Stepglm[both]+Enet[alpha=0.3] | CD34, SIRT6, C3, CCR7, CCL19, THBS1 |
| Stepglm[both]+Enet[alpha=0.4] | CD34, SIRT6, C3, CCR7, CCL19, THBS1 |
| Stepglm[both]+Enet[alpha=0.5] | CD34, SIRT6, C3, CCR7, CCL19, THBS1 |
| Stepglm[both]+Enet[alpha=0.6] | CD34, SIRT6, C3, CCR7, CCL19, THBS1 |
| Stepglm[both]+Enet[alpha=0.7] | CD34, SIRT6, C3, CCR7, CCL19, THBS1 |
| Stepglm[both]+Enet[alpha=0.8] | CD34, SIRT6, C3, CCR7, CCL19, THBS1 |
| Stepglm[both]+Enet[alpha=0.9] | CD34, SIRT6, C3, CCR7, CCL19, THBS1 |
| Stepglm[both]+GBM | THBS1, CD34, CCL19, C3, SIRT6, CCR7 |
| Stepglm[both]+glmBoost | CD34, SIRT6, C3, CCR7, CCL19, THBS1 |
| Stepglm[both]+Lasso | CD34, SIRT6, C3, CCR7, CCL19, THBS1 |
| Stepglm[both]+LDA | CD34, SIRT6, C3, CCR7, CCL19, THBS1 |
| Stepglm[both]+NaiveBayes | CD34, SIRT6, C3, CCR7, CCL19, THBS1 |
| Stepglm[both]+plsRglm | CD34, SIRT6, C3, CCR7, CCL19, THBS1 |
| Stepglm[both]+Ridge | CD34, SIRT6, C3, CCR7, CCL19, THBS1 |
| Stepglm[both]+SVM | CD34, SIRT6, C3, CCR7, CCL19, THBS1 |
| Stepglm[forward] | LILRA2, ISL1, CD34, EMILIN1, AEBP1, RB1, S1PR1, NOTCH1, SIRT6, CD96, CD226, CD160, HLA_E, HLA_F, HLA_G, CDK5, CX3CR1, EPHA4, DKK1, ITGAM, TREM2, CX3CL1, C1QA, C1QB, C3, LILRB1, FGL2, `HLA-DMB`, ICAM1, ITGAL, LGALS9, APBB1IP, CD81, NOD1, CCR7, FCGR2B, CLEC4A, CCL19, CCL21, NOD2, SLC11A1, THBS1, CD68, CD74, EFNB2, MDK, COL1A1, COL4A1, COL4A2, COL4A3, COL4A4, COL4A5, COL4A6, DDR2, SYK, DDR1, HLA_DMA, HLA_DRA, TAPBPL, TAPBP, CALR, C1QL1, ANKS1A, RND1, APP, BCL11A, SCARF1, FARP2, MSN, JAM2, RAC2, STK10, ZAP70, EPHA2, VASH1, VEGFC, GLCE, ANGPT1, NDST1, IDUA, CSGALNACT1, XYLT2, NDST4, NDST2, NDST3, TMSB15A, TWF2, GSN, SCIN, TMSB4Y, TMSB10, GJA1, ARHGDIB, TIMP1, DOCK2, MAL, PTPRC, RALA, CD2, MICA, PIBF1, FGR, RHBDD3, PGLYRP4, PGLYRP1 |
| SVM | LILRA2, ISL1, CD34, EMILIN1, AEBP1, RB1, S1PR1, NOTCH1, SIRT6, CD96, CD226, CD160, HLA_E, HLA_F, HLA_G, CDK5, CX3CR1, EPHA4, DKK1, ITGAM, TREM2, CX3CL1, C1QA, C1QB, C3, LILRB1, FGL2, HLA-DMB, ICAM1, ITGAL, LGALS9, APBB1IP, CD81, NOD1, CCR7, FCGR2B, CLEC4A, CCL19, CCL21, NOD2, SLC11A1, THBS1, CD68, CD74, EFNB2, MDK, COL1A1, COL4A1, COL4A2, COL4A3, COL4A4, COL4A5, COL4A6, DDR2, SYK, DDR1, HLA_DMA, HLA_DRA, TAPBPL, TAPBP, CALR, C1QL1, ANKS1A, RND1, APP, BCL11A, SCARF1, FARP2, MSN, JAM2, RAC2, STK10, ZAP70, EPHA2, VASH1, VEGFC, GLCE, ANGPT1, NDST1, IDUA, CSGALNACT1, XYLT2, NDST4, NDST2, NDST3, TMSB15A, TWF2, GSN, SCIN, TMSB4Y, TMSB10, GJA1, ARHGDIB, TIMP1, DOCK2, MAL, PTPRC, RALA, CD2, MICA, PIBF1, FGR, RHBDD3, PGLYRP4, PGLYRP1 |
