# Supplementary material for: A nine-gene diagnostic model for IgA nephropathy based on multi-cohort machine learning: integrating gene expression and immunohistochemical validation
Source: Ren Fail. 2026 Mar 9;48(1):2637355. doi: 10.1080/0886022X.2026.2637355 (PMC12978185; doi:10.1080/0886022X.2026.2637355)
Supplement: Supplementary Table 6.docx [file IRNF_A_2637355_SM7517.docx]

**Supplementary Table 6. Nine-Gene Panel Summary: Biological Functions and Representative Pathways.**

| Gene | Biological function (concise) | Representative pathways |
| --- | --- | --- |
| CD160 | Coinhibitory/immune-checkpoint receptor on NK and T cells; modulates cytotoxicity and T-cell activation | TNF-receptor superfamily signaling; immune regulation |
| CX3CR1 | Receptor for fractalkine (CX3CL1); mediates leukocyte adhesion, migration, and tissue patrolling | Chemokine signaling; leukocyte trafficking; renal inflammation |
| EPHA4 | Eph receptor tyrosine kinase; governs cell motility, axon guidance, and tissue patterning | Eph/ephrin signaling; axon guidance; cytoskeletal dynamics |
| THBS1 | Matricellular glycoprotein; activates latent TGF-β; signals via CD47 to inhibit angiogenesis | TGF-β activation; CD47 signaling; extracellular matrix/angiogenesis |
| HLA-DRA | MHC class II α-chain; essential for antigen processing/presentation to CD4⁺ T cells | Antigen processing and presentation; adaptive immune response |
| FARP2 | RhoGEF activating Rac1/Cdc42; controls cytoskeletal remodeling and neurite/filopodia dynamics | Rho-GTPase signaling; axon guidance; cell polarity |
| VASH1 | Endothelium-derived anti-angiogenic factor; measurable in plasma/urine | Angiogenesis regulation; endothelial homeostasis |
| TMSB4Y | Y-linked thymosin-β family member; actin monomer binding and cytoskeleton organization | Actin cytoskeleton organization; cell migration |
| RHBDD3 | Rhomboid-like regulator restraining dendritic-cell activation and IL-6 production | TLR–NF-κB/ubiquitin signaling; innate immune regulation |
